# p38MAP kinase regulates senescence in human iPS-derived myocytes

**DOI:** 10.18632/aging.206385

**Published:** 2026-05-28

**Authors:** Hiroki Sato, Momoka Arakane, Yuto Mizuno, Takashi Sasaki, Hidetoshi Sakurai, Masamichi Kamihira, Atsushi Enomoto, Kazuaki Takafuji, Junichiro Takaya, Toshihide Suzuki, Yu Takahashi, Yoshio Yamauchi, Ryuichiro Sato, Makoto Shimizu

**Affiliations:** 1 Nutri-Life Science Laboratory, Department of Applied Biological Chemistry, Graduate School of Agricultural and Life Sciences, The University of Tokyo, Tokyo 113-8657, Japan; 2 Laboratory of Nutrition and Life Science, Graduate School of Humanities and Sciences, Ochanomizu University, Tokyo 112-8610, Japan; 3 Center for iPS Cell Research and Application, Kyoto University, Kyoto 606-8507, Japan; 4 Department of Chemical Engineering, Faculty of Engineering, Kyushu University, Fukuoka 819-0395, Japan; 5 Laboratory of Molecular Radiology, Center for Disease Biology and Integrative Medicine, Graduate School of Medicine, The University of Tokyo 113-0033, Japan; 6 Research Institute, Suntory Global Innovation Center, Ltd., Kyoto 619-0284, Japan; 7 Laboratory of Food Biochemistry, Department of Applied Biological Chemistry, Graduate School of Agricultural and Life Sciences, The University of Tokyo, Tokyo 113-8657, Japan; 8 Institute for Human Life Innovation, Ochanomizu University, Tokyo 112-8610, Japan

**Keywords:** senescence, skeletal muscle, iPS cell, p38MAP kinase, DNA damage

## Abstract

Skeletal muscle displays an age-associated decline in motor function and muscle mass. However, the precise mechanisms underlying skeletal muscle aging, particularly within muscle fibers, remain poorly understood. Here, we demonstrate that DNA damage induced by X-ray irradiation triggers a senescence-like phenotype in human iPS cell (iPSC)-derived myocytes. This irradiation leads to muscle fiber atrophy and reduced contractile activity, accompanied by elevated expression of the aging marker p21. Omics analyses revealed that DNA damage activates p38 mitogen-activated protein kinase (p38MAPK). Inhibition of p38MAPK mitigated the senescence-like phenotype. These results suggest that activation of p38MAPK plays a role in regulating skeletal muscle senescence and that DNA damage-induced senescence in iPSC-derived myocytes represents a viable model for studying human skeletal muscle senescence.

## INTRODUCTION

The global population is aging at an unprecedented rate, resulting in a rapidly growing elderly demographic [1]. A major concern in this aging society is the rising incidence of age-related diseases, including cardiovascular disorders, neurodegenerative conditions, metabolic syndromes, and sarcopenia. Sarcopenia is a progressive, age-related skeletal muscle disorder characterized by accelerated loss of muscle mass and function, which increases the risk of falls, fractures, and frailty [2].

Cellular senescence is widely recognized as a fundamental driver of aging and age-related diseases. In proliferating cells, senescence is induced by irreversible cell cycle arrest in response to various stressors such as DNA damage and oxidative stress and is associated with the activation of cell cycle inhibitors p16 and p21 [3]. These senescent cells contribute to chronic inflammation through the senescence-associated secretory phenotype (SASP). Their persistent presence has been linked to tissue dysfunction, frailty, and several age-associated conditions, making them an important target for therapeutic intervention.

In recent years, several studies have suggested a link between cellular senescence and individual aging, indicating that the elimination of senescent cells (senolysis) and modulation of the senescence-associated secretome (senostatics) may be promising strategies for extending healthspan [4]. Senescent cells exhibit distinct phenotypes, including activation of p38 mitogen-activated protein kinase (p38MAPK) and the SASP [5]. The integrin–SRC pathway has been identified as an upstream regulator of p38MAPK signaling [6], suggesting it plays a role in the regulation of senescence. To date, aging research has largely focused on proliferating cells, with limited attention given to differentiated, nonproliferating cells. Skeletal muscle consists of myofibers and other components including myogenic precursor cells (muscle satellite cells; MuSCs) and multipotent precursor cells (mesenchymal stem cells; MSCs). Aging-related changes have been reported in both MuSCs and MSCs. In the skeletal muscle of aged or senescence-accelerated mice, the number of myocyte and muscle regeneration capacity are reduced. MuSCs isolated from aged mice express elevated levels of the aging marker p16 and exhibit irreversible proliferative arrest [7]. Similarly, MSCs undergo cellular senescence with aging, as evidenced by a decline in their numbers, proliferative capacity, and contribution to muscle regeneration in aged mice [8]. In contrast to these precursor cells, myocytes—a key component of myofibers—have been less extensively studied. Skeletal muscle is frequently subjected to oxidative stress, particularly during exercise, which generates reactive oxygen species [9, 10]. Notably, myocytes are thought to have an exceptionally long lifespan, estimated to be around 50 years [11]. Although the mechanisms of skeletal muscle fiber aging remain poorly understood, studies have reported age-related changes in gene expression in both humans and mice [12, 13]. Transcriptome analysis of single myofibers has revealed increased expression of the aging marker p21 in aged mouse skeletal muscle. Further investigations have shown that p53 signaling, cytokine–cytokine interaction, and TGF-β signaling pathways are upregulated in the muscle fibers of aged mice with high p21 expression. These findings suggest that p21, commonly used as a marker of cellular senescence, may also serve as a marker for skeletal muscle fiber senescence. This evidence motivates further investigation into the mechanisms underlying myofiber aging.

The mechanism of skeletal muscle aging remains poorly understood, partly due to the lack of an *in vitro* system for assessing the function of differentiated myocytes. Previous studies have shown that DNA-damaging agents, such as X-ray irradiation, can induce cellular senescence in proliferating cells and in mice. In this study, we applied X-ray irradiation to differentiated human myocytes, specifically hiPSC-myocytes. We demonstrated that X-ray irradiation induces senescence-like phenotypes in hiPSC-myocytes, as evidenced by increased expression of senescence-related genes, myotube atrophy, and reduced contractile capacity. Further analysis revealed that X-ray-induced senescence occurs via activation of p38MAPK, suggesting that the integrin-p38MAPK signaling pathway is a contributing factor in skeletal muscle senescence.

## RESULTS

### DNA damage induces cellular senescence in hiPSC-derived myocytes

In an effort to establish a senescence model of human myocytes, iPSC-derived myocytes were exposed to genotoxic agents such as X-rays and H_2_O_2_. Preliminary experiments indicated that X-ray irradiation at 3 Gy induced DNA damage without causing acute cell death (data not shown). We utilized the hiPSC 409B2^tet-MyoD^, a cell line which harbors tetracycline-inducible human MYOD1 expression [14] and acquires contractile activity through EPS [15, 16]. On day 6 of myogenic differentiation, hiPSCs were irradiated with X-rays at 3 Gy and subsequently cultured for one week. Given that muscle atrophy and functional decline are key features of skeletal muscle aging, we analyzed the myofiber structure and contractile function of the hiPSCs. Immunostaining for MHC revealed a significant reduction in the MHC-positive area following X-ray irradiation (Figure 1A). Motion analysis using EPS demonstrated that contractile parameters, including myotube displacement and contraction velocity, were also significantly reduced (Figure 1B). The expression of senescence markers was altered: p21 was upregulated, whereas Lamin B1 was downregulated (Figure 1C). Consistent results were also observed at the protein level (Figure 1D). p16 expression showed a slight increase at the protein level (Figure 1D). Although induction of γH2AX, a marker of DNA damage, subsided within 24 h (Figure 1E), p21 and Lamin B1 levels remained at both mRNA and protein levels up to day 7 post-irradiation (Figure 1C, 1D). Comparable outcomes were observed with doxorubicin, another DNA-damaging agent (Supplementary Figure 1A–1C). These findings suggest that DNA damage induces cellular senescence-phenotype in hiPSC-derived myocytes.

**Figure 1:**
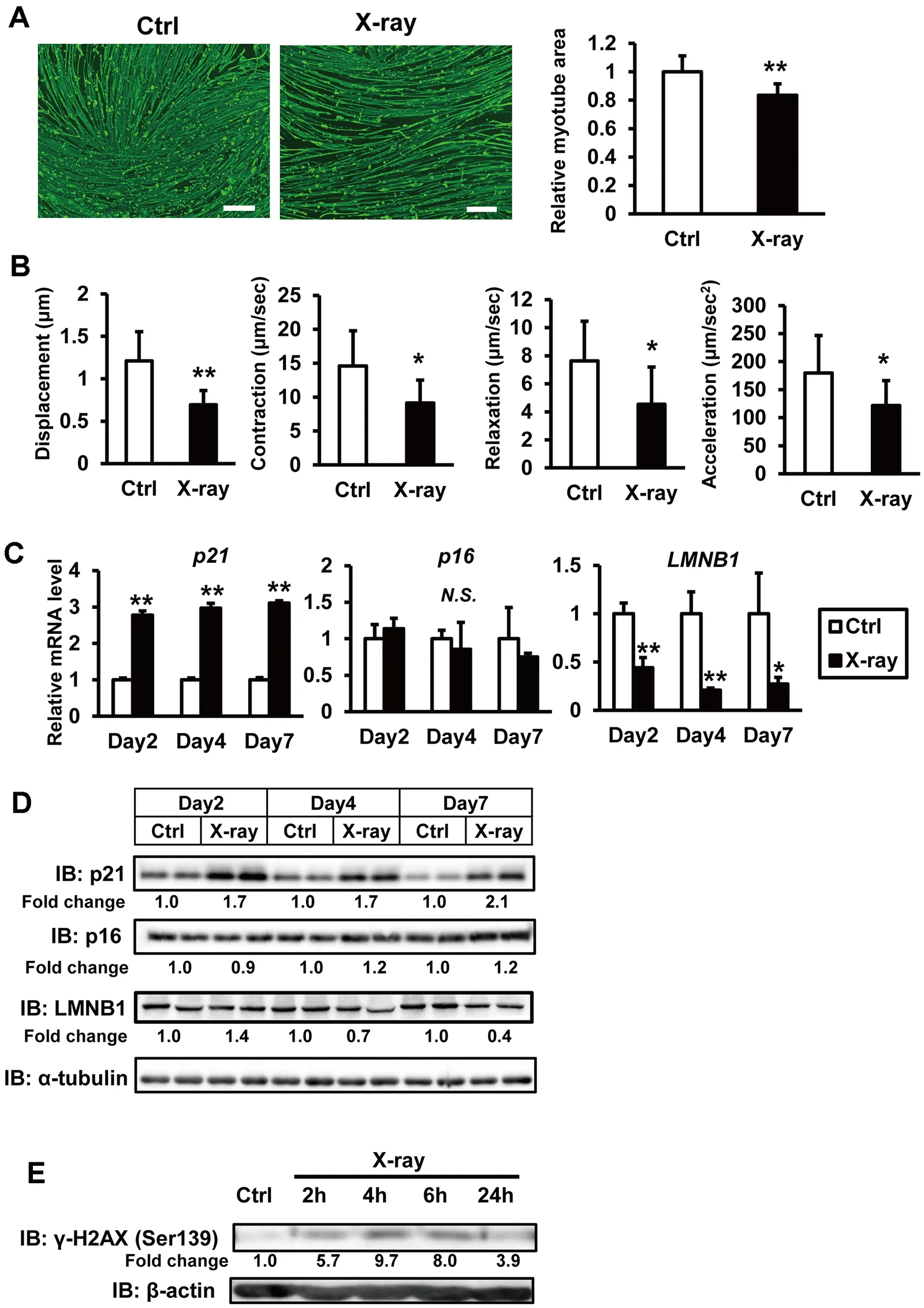
**X-ray irradiation induces senescence in hiPSC-derived myocytes.** (**A**) hiPSCs 409B2^tet-MyoD^ were differentiated into myocytes and irradiated with X-rays (3 Gy). One week post-irradiation, cells were fixed, permeabilized, and stained for MHC and nuclei. Scale bar: 100 μm. Relative MHC-positive areas were quantified as described in the methods section. The relative myotube area in nonirradiated controls (Ctrl) was set to 1. (**B**) hiPSC-derived myocytes were stimulated with electrical pulses (23 V, 4 ms, 1 Hz). Myotube movement was measured using motion analysis software. (**C**) hiPSC-derived myocytes were irradiated with X-rays (3 Gy), and total RNA was isolated one week later. mRNA levels were measured by real-time PCR and normalized to 18S rRNA. Relative mRNA expression in nonirradiated controls (Ctrl) was set to 1. (**D**, **E**) Whole-cell lysates from hiPSC-derived myocytes were collected at the indicated time points after X-ray irradiation and subjected to immunoblotting to assess the expression of the indicated proteins. The numbers under the lanes indicate fold changes in protein level relative to the control and were standardized against α-tubulin or β-actin. Data are presented as mean ± SD (n = 3 or 9). Statistical significance was determined using Student’s t-test. *p < 0.05, **p < 0.01.

### DNA damage increases SASP factors

The SASP, in which senescent cells produce a variety of secreted proteins, including inflammatory cytokines, is a hallmark of senescence. To investigate the senescence secretome of irradiated hiPSC-myocytes, quantitative shotgun proteomics was performed. This analysis revealed that 65 proteins were elevated seven days after X-ray irradiation (Supplementary Table 2), and their levels gradually increased post-irradiation (Figure 2A). Notably, several proteins such as FN1 and CYR61 were detected in the medium of irradiated hiPSC-myocytes. To annotate the identified secreted proteins, we classified them based on Gene Ontology (GO) using the integrative DAVID platform [17, 18]. The results showed that proteins associated with “extracellular matrix constituent” and “cytokine activity” in the molecular function category were highly enriched (Figure 2B). Notably, several proteins detected in the proteomics analysis have been reported as SASP factors. Furthermore, RNA analysis confirmed that the expression of several of these genes was significantly upregulated following X-ray irradiation (Figure 2C). Western blot analyses revealed an increase in secreted FN1 and CYR61 proteins in the culture medium by X-ray irradiation (Figure 2D). Similar results were observed in hiPSC-myocytes treated with doxorubicin (Supplementary Figure 1D). These findings demonstrate that DNA damage of hiPSC-myocytes leads to increased expression and secretion of SASP factors.

**Figure 2:**
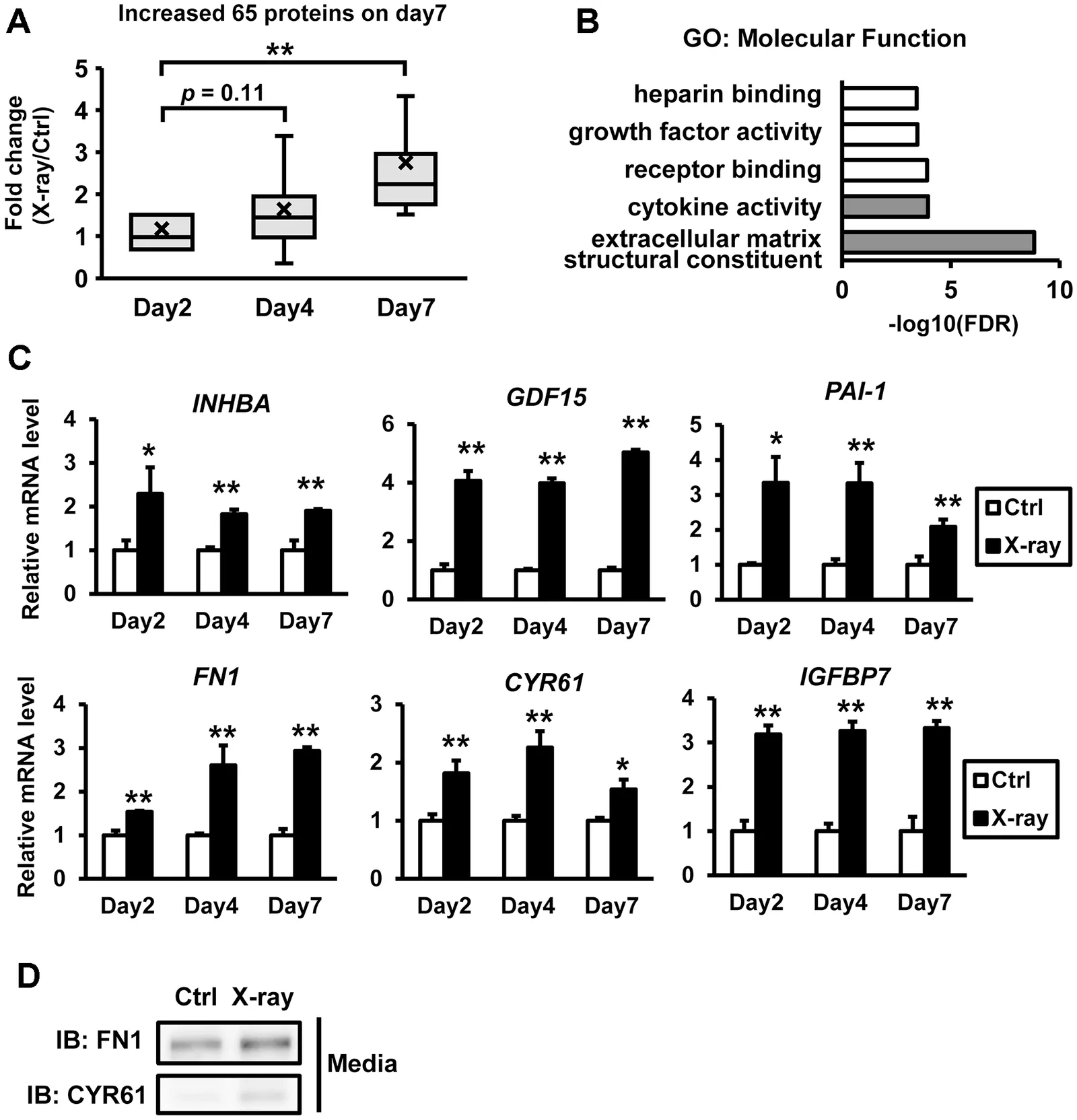
**Comprehensive proteomic profiling of the senescence-associated secretome in hiPSC-derived myocytes.** Proteomic analyses were performed using conditioned media from irradiated hiPSC-derived myocytes. A total of 65 proteins were found to be upregulated on day 7 post X-ray irradiation. (**A**) Distribution of the 65 upregulated proteins. (**B**) Gene Ontology (GO) analysis of the secreted proteins in the myocyte-conditioned media, focused on molecular function. The top five GO terms are listed. (**C**) Total RNA was isolated at the indicated time points following X-ray irradiation. mRNA levels were quantified by real-time PCR and normalized to 18S rRNA. Relative mRNA expression levels in nonirradiated controls (Ctrl) were set to 1. (**D**) Culture media from hiPSC-derived myocytes were collected at the indicated time points after X-ray irradiation and subjected to immunoblotting to assess the expression of the indicated proteins. Data are presented as mean ± SD (n = 3). Statistical significance was assessed using Student’s t-test. *p < 0.05, **p < 0.01. Abbreviations: INHBA: inhibin subunit beta A; GDF15: growth differentiation factor 15; PAI-1: serpin family E member 1; FN1: fibronectin 1; CYR61: cellular communication network factor 1; IGFBP7: insulin like growth factor binding protein 7.

### p38MAPK signaling is activated by DNA damage

Proteomics and GO analyses revealed that several proteins (FN1, CYR61, and CTGF) classified under the term “extracellular matrix structural constituent” were upregulated following X-ray irradiation. These proteins are known ligands for integrins, which regulate cell–extracellular matrix (ECM) adhesion and cytokine receptor signaling. Recent studies have shown that integrin signaling plays a role in regulating cellular senescence in fibroblasts [19]. To determine whether X-ray irradiation induces the expression of integrin family genes, we examined mRNA levels of integrin subunits. The expression of integrin subunit genes such as ITGA4 and ITGA5 was significantly increased in parallel with the upregulation of FN1, CYR61, and CTGF mRNA following irradiation, suggesting an important role of integrin signaling on our senescence model (Figure 3A). It has been reported that p38MAPK and SRC/FAK are key mediators of integrin–induced signaling [20] and that senescence triggers p38MAPK phosphorylation via the SRC/FAK pathway [19]. These findings led us to investigate the involvement of p38MAPK and SRC/FAK signaling in our senescence model of human myocytes. Western blot analysis showed a gradual increase in p38MAPK phosphorylation after X-ray irradiation (Figure 3B). Similar results were observed in hiPSC-myocytes treated with doxorubicin (Supplementary Figure 1B). Pharmacological inhibition of SRC and FAK using AZD0530 and GSK2256098, respectively, reduced p38MAPK phosphorylation (Figure 3C, 3D). These results indicate that the integrin–FAK/SRC–p38MAPK signaling axis is activated during senescence in hiPSC-derived myocytes.

**Figure 3:**
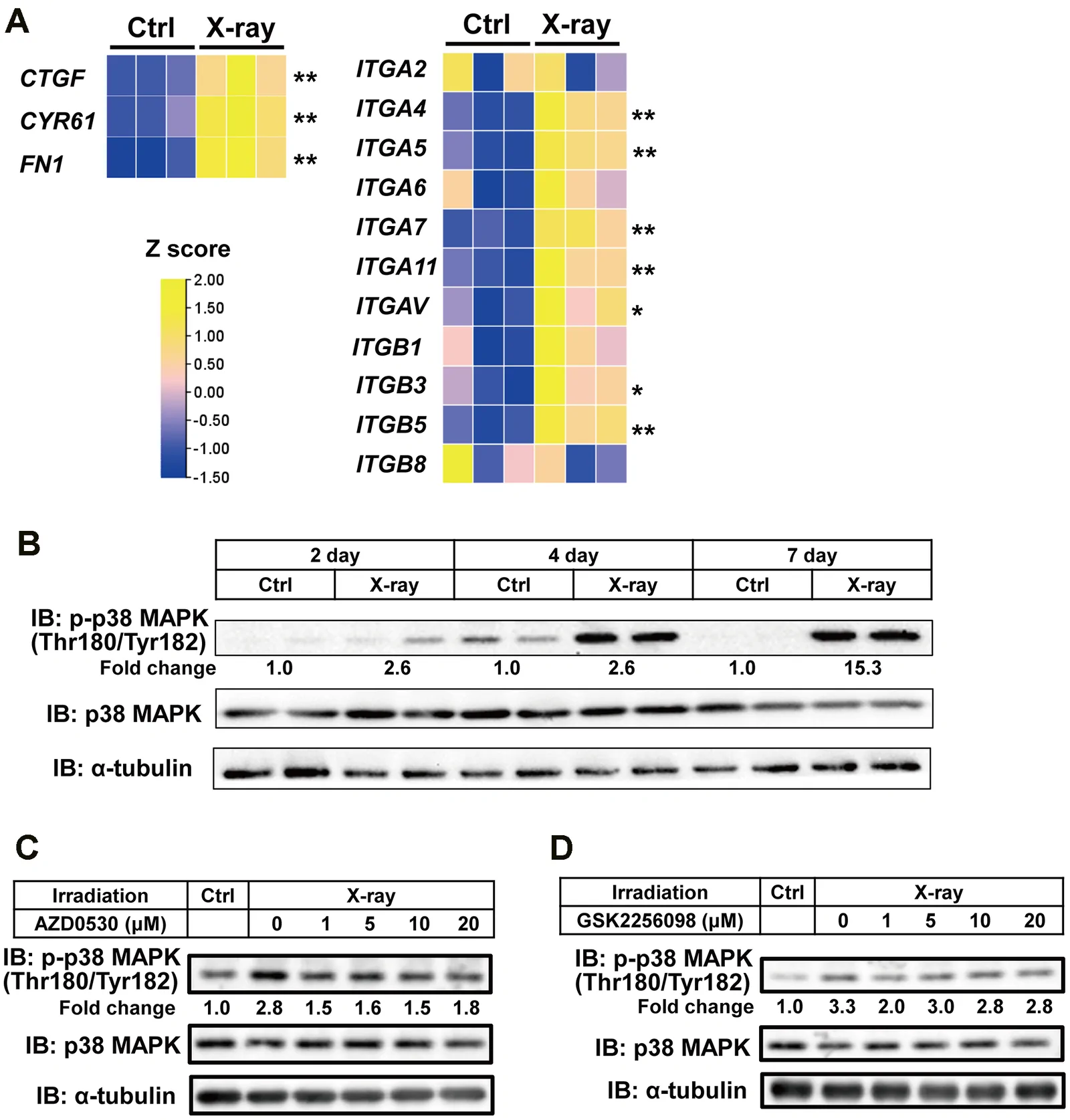
**Activation of p38MAPK in senescent hiPSC-derived myocytes.** (**A**) Representative mRNA expression data from irradiated myocytes. One week after X-ray irradiation, total RNA was extracted and analyzed by real-time PCR. Expression levels were normalized to 18S rRNA and presented as relative values. (**B**–**D**) Whole-cell lysates from hiPSC-derived myocytes were collected at the indicated time points post X-ray irradiation. Lysates were subjected to immunoblotting to assess the expression of the indicated proteins. The numbers under the lanes indicate fold changes in protein level relative to the control and were standardized against total p38MAPK.

### p38MAPK mediates DNA damage-induced senescence in hiPSC-myocytes

We next investigated whether p38MAPK mediates irradiation-induced senescence in hiPSC-myocytes. Preliminary experiments showed that treatment with two p38MAPK inhibitors (SB202190 and SB203580) reduced the phosphorylation of HSP27, a known substrate of p38MAPK (Supplementary Figure 2), indicating attenuation of p38MAPK signaling in hiPSC-myocytes. Immunostaining for MHC revealed that irradiation-induced myofiber atrophy was significantly alleviated by p38MAPK inhibition (Figure 4A). Motion analysis using EPS further demonstrated partial restoration of contractile activity (Figure 4B). A similar recovery of contractile function with p38MAPK inhibitors was observed in hiPSC-myocytes treated with doxorubicin (Supplementary Figure 3A). To explore the potential crosstalk between p38MAPK signaling and skeletal muscle senescence, transcriptome analysis was performed. RNA sequencing revealed that X-ray irradiation caused significant alterations in gene expression, which were partially reversed by p38MAPK inhibition (Figure 4C and Supplementary Table 3). Further analysis identified 1,118 genes regulated by irradiation through p38MAPK signaling. GO analysis showed that genes associated with “extracellular matrix structural constituent” and “cytokine activity” were highly enriched (Figure 4D), consistent with the proteomics data (Figure 2B). The expression of genes within these enriched categories was further examined by quantitative PCR. Some irradiation-induced genes such as INHBA were downregulated in the presence of p38MAPK inhibitors (Figure 4E). Similar gene expression trends were observed in doxorubicin-treated cells (Supplementary Figure 3B). These findings demonstrate that p38MAPK activation contributes to the upregulation of SASP factors in hiPSC-derived myocytes. To further elucidate the role of p38MAPK, we performed analyses using p38MAPK activators. Because no direct activators of p38MPK have been reported, we searched for compounds which activate p38MAPK in hiPSC-derived myocytes. In preliminary experiments searching potential p38MAPK activating drugs [21, 22], treatments of anisomycin or arsenite increased p38MAPK phosphorylation in hiPSC-derived myocytes (data not shown). When hiPSC-derived myocytes were cultured with anisomycin or arsenite for 5 days, both drugs increased p38MAPK phosphorylation (Figure 4F, 4G). and decreased myotube area (Figure 4H, 4I). These results further support our hypothesis that p38MAPK plays an important role in the senescence-like phenotype of hiPSC-derived myocytes. We next investigated the effects of senolytic drug on p38MAPK signaling and senescence-like phenotypes in hiPSC-derived myocytes. Treatment of dasatinib, a senolytic drug [23], reduced X-ray irradiation–induced phosphorylation of p38MAPK (Figure 4J). Contrary to our expectations, irradiation-induced myofiber atrophy was significantly alleviated by dasatinib treatment (Figure 4K, 4L) without affecting myotube number (Supplementary Figure 4). Similar results were observed in hiPSC-derived myocytes treated with doxorubicin (Supplementary Figure 5A, 5B). Because dasatinib is also known as a SRC inhibitor, it may improve myofiber phenotypes by inhibiting SRC–p38MAPK signaling. Finally, we performed Gene Set Enrichment Analysis (GSEA) [25] to investigate functional gene enrichment in human skeletal muscle [26]. GSEA revealed age-related upregulation of gene transcripts associated with “MAPK signaling,” “focal adhesion,” and “cytokine–cytokine receptor interaction” pathways (Figure 4M). These findings highlight the significant roles of MAPK and integrin signaling during senescence of human skeletal muscle.

**Figure 4:**
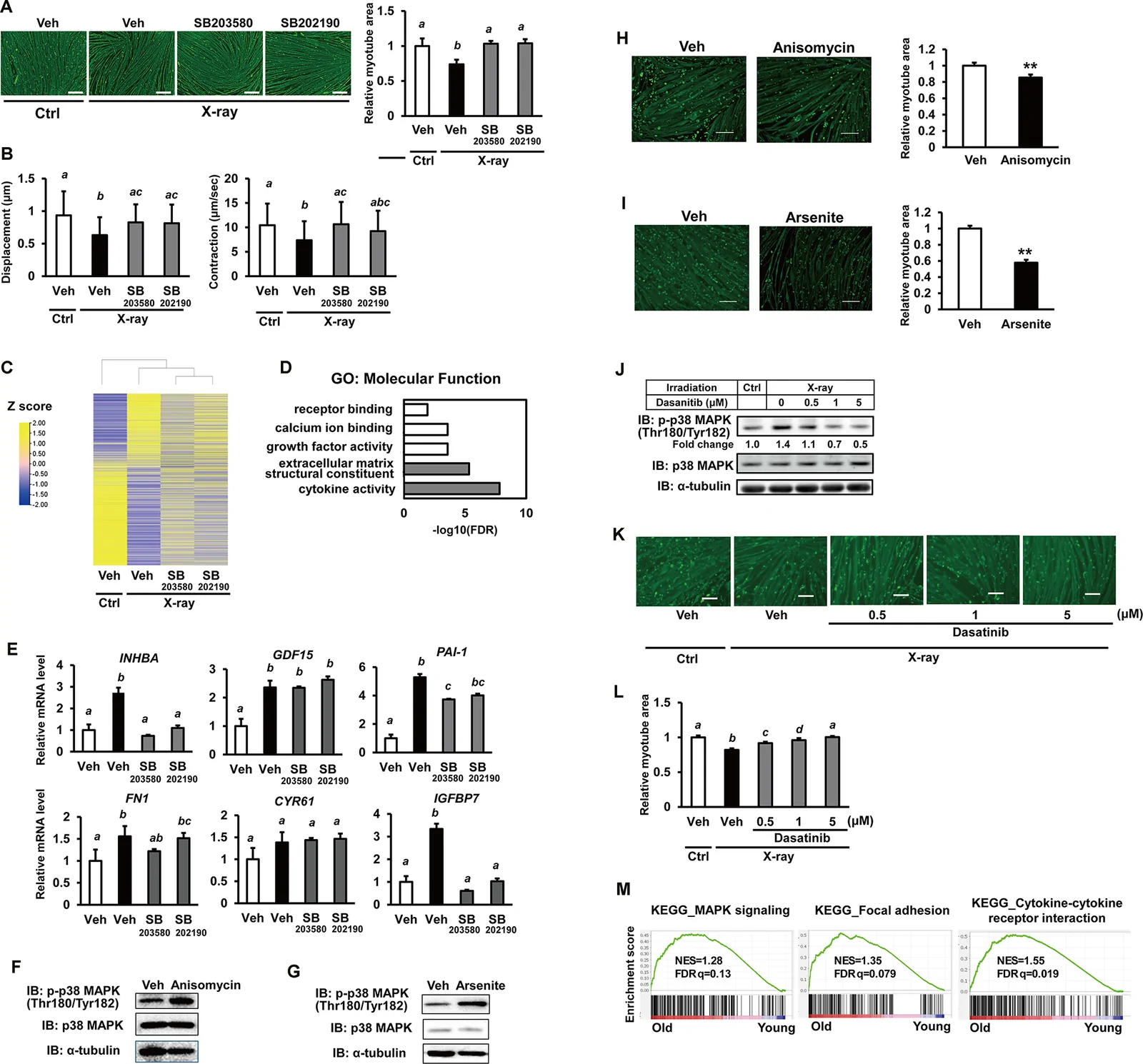
**Senescence in hiPSC-derived myocytes is mediated via the p38MAPK pathway.** (**A**) hiPSC-derived myocytes were irradiated with X-rays (3 Gy). One day post-irradiation, cells were treated with p38MAPK inhibitors (SB203580 and SB202190). One week after irradiation, cells were stained for MHC and nuclei. Scale bar: 100 μm. Relative MHC-positive areas were quantified as described in the methods section. (**B**) hiPSC-derived myocytes were stimulated with electrical pulses (23 V, 4 ms, 1 Hz), and myotube movement was measured using motion analyzer software. (**C**) Comprehensive transcriptome analysis of irradiated myocytes treated with or without p38MAPK inhibitors. (**D**) Gene Ontology (GO) analysis of molecular function in myocytes. The top five GO terms are listed. (**E**) One week after X-ray irradiation, total RNA was extracted and analyzed by real-time PCR. mRNA levels were normalized to 18S rRNA. Relative mRNA expression in untreated, nonirradiated controls (Ctrl) was set to 1. (**F**, **G**) Whole-cell lysates from hiPSC-derived myocytes were collected on 5 days after anisomycin or arsenite treatment and subjected to immunoblotting to assess the expression of the indicated proteins. (**H**, **I**) 5 days after anisomycin or arsenite treatment, cells were fixed, permeabilized, and stained for MHC and nuclei. Scale bar: 100 μm. Relative MHC-positive areas were quantified as described in the methods section. The relative myotube area in nonirradiated vehicles (Veh) was set to 1. (**J**) One week after X-ray irradiation, iPSC-derived myocytes were treated with dasatinib for 6hrs. Whole-cell lysates from hiPSC-derived myocytes were collected subjected to immunoblotting to assess the expression of the indicated proteins. (**K**, **L**) One week after X-ray irradiation and dasatinib treatment, cells were fixed, permeabilized, and stained for MHC and nuclei. Scale bar: 100 μm. Relative MHC-positive areas were quantified as described in the methods section. The relative myotube area in nonirradiated vehicles without X-ray irradiation was set to 1. (**M**) Gene Set Enrichment Analysis (GSEA) of the indicated KEGG pathways. KEGG: Kyoto Encyclopedia of Genes and Genomes; NES: normalized enrichment score; FDR: false discovery rate. The image in Figure 4 was generated using TBtools [24]. The numbers under the lanes indicate fold changes in protein level relative to the control and were standardized against total p38MAPK. Data are presented as mean ± SD (n = 3 or 27), and statistical analysis was performed using Tukey’s test. Different letters indicate significant differences between groups (p < 0.05).

## DISCUSSION

Our current study demonstrated that *in vitro* human skeletal muscle models of senescence can be established by exposing iPSC-derived myocytes to DNA-damaging agents such as X-rays. We identified the integrin–p38MAPK pathway as a key regulatory axis of skeletal muscle senescence in this model, suggesting that similar signaling may be activated in skeletal muscle, as observed in proliferative cells commonly used in aging research. While the link between the integrin–p38MAPK pathway and cellular senescence has been previously reported—particularly in studies where irradiation or DNA-damaging agents activate this pathway in fibroblasts—it remains to be fully elucidated [5, 19]. Aging research has traditionally focused on proliferating cells, with relatively few studies addressing terminally differentiated, non-proliferating cells. This study shows that a comparable pathway is engaged during senescence in such differentiated cells. Expression of the DNA damage marker γH2AX was detected in the early phase (Figure 1E), whereas SASP factors secretion and other senescence-associated proteins gradually increased after X-ray irradiation (Figure 2A). These findings suggest that senescence in this model arises during the post-damage incubation period, rather than during the acute phase of DNA damage. Notably, inhibition of p38MAPK suppressed the senescence-like phenotype induced by X-ray irradiation (Figure 4). Proteomics and GO analyses revealed that pathways associated with “extracellular matrix structural constituent” and “cytokine activity” were highly enriched (Figure 2B), prompted us to further investigate these pathways. In contrast, X-ray irradiation also activated other pathways such as growth factor activity (Figure 2B). Although p38MAPK inhibitors rescued the senescence-like phenotypes—suggesting that p38MAPK is a major signaling pathway in this context—our results do not exclude contributions from these other pathways. The expression of a limited set of SASP factors, including INHBA and PAI-1, was decreased by p38MAPK inhibitors, even though myotube atrophy was rescued. This observation suggests two possible mechanisms. First, p38MAPK is an important signaling pathway but may not be the master regulator of SASP factor expression. Second, some SASP factors reduced by p38MAPK inhibition may contribute to myofiber homeostasis. In particular, INHBA and PAI-1 have been reported to be involved in muscle atrophy [27, 28]. Phosphorylation of p38MAPK has also been observed in aging human skeletal muscle [29]. Moreover, studies using muscle-specific p38MAPK knockout mice have shown that p38MAPK is important for skeletal muscle homeostasis during aging and in the context of muscle atrophy [30]. These findings support a potential link between this signaling pathway and skeletal muscle aging. Senolytic drug dasatinib in general induces cell clearance in senescent cells; however, in this evaluation system, iPSC-myotubes were treated with during the process of acquiring a senescent phenotype. Although MTT assay, which is commonly used for cell counting, assesses mitochondrial activity, mitochondrial activity in skeletal muscle is known to change dynamically in response to various stimuli. We therefore used myotube number to obtain a more accurate assessment. Consequently, no senescent cell clearance effect was observed. Instead, it may function as a SRC inhibitor, suppressing downstream p38MAPK and inhibiting senescence induction. While pro-apoptotic senolytic drugs, such as ABT-737 and navitoclax, may be effective, our results showed no changes in apoptotic pathways in our senescence model (data not shown). Ideally, fully aged myocytes should be examined to determine whether senolytic drugs eliminate these cells. However, since skeletal muscle remodeling generally requires a long time, functional recovery, including muscle contractile activity, is expected to take even longer. Therefore, our experimental system of iPSC-myotubes has limitations due to the impossibility of long-term culture. On the other hand, our findings raise the possibility that senolytic drugs may exert distinct effects during the early stages of senescence, potentially ameliorating senescence-associated phenotypes rather than inducing cell elimination.

Skeletal muscle comprises various cell populations beyond the myocytes investigated in this study. For instance, MSCs are integral components of skeletal muscle and have been reported to exhibit cellular inflammation; notably, the removal of senescent MSCs using senolytic drugs has been shown to restore cellular function [31]. MuSCs, which are essential for muscle regeneration, have also been observed to develop a senescence-like phenotype [32]. Abnormal activation of the integrinβ1-MAPK pathway is one proposed mechanism underlying the functional decline of these cells during aging, leading to impaired tissue regeneration [33]. In the present study, we found that senescence also activates the integrin-p38MAPK pathway in myotubes, yielding similar results (Figure 3). However, it has been reported that decreased fibronectin and destabilization of stem cell adhesion in the context of age-related ECM remodeling may lead to decreased muscle regeneration. Future studies of the relationship between MuSCs, MSCs, and myocytes in aged skeletal muscle should be investigated. Since the *in vitro* model described here involved the culture of myocytes alone, it did not allow analysis of cellular interactions with other skeletal muscle components. In order to analyze the aging of skeletal muscle, experiments using transwell in coculture systems can be considered.

In summary, we developed a system to induce senescence in human myocytes by irradiating hiPSC-derived myocytes. Using this established evaluation model, we demonstrated that SASP secretion is enhanced during senescence, similar to what is observed in aging proliferative cells. Moreover, our findings suggest that activation of the integrin/p38MAPK pathway plays a regulatory role in skeletal muscle senescence (Figure 5). These results indicate that targeting this pathway may offer a promising strategy to mitigate age-related functional decline of skeletal muscle.

**Figure 5:**
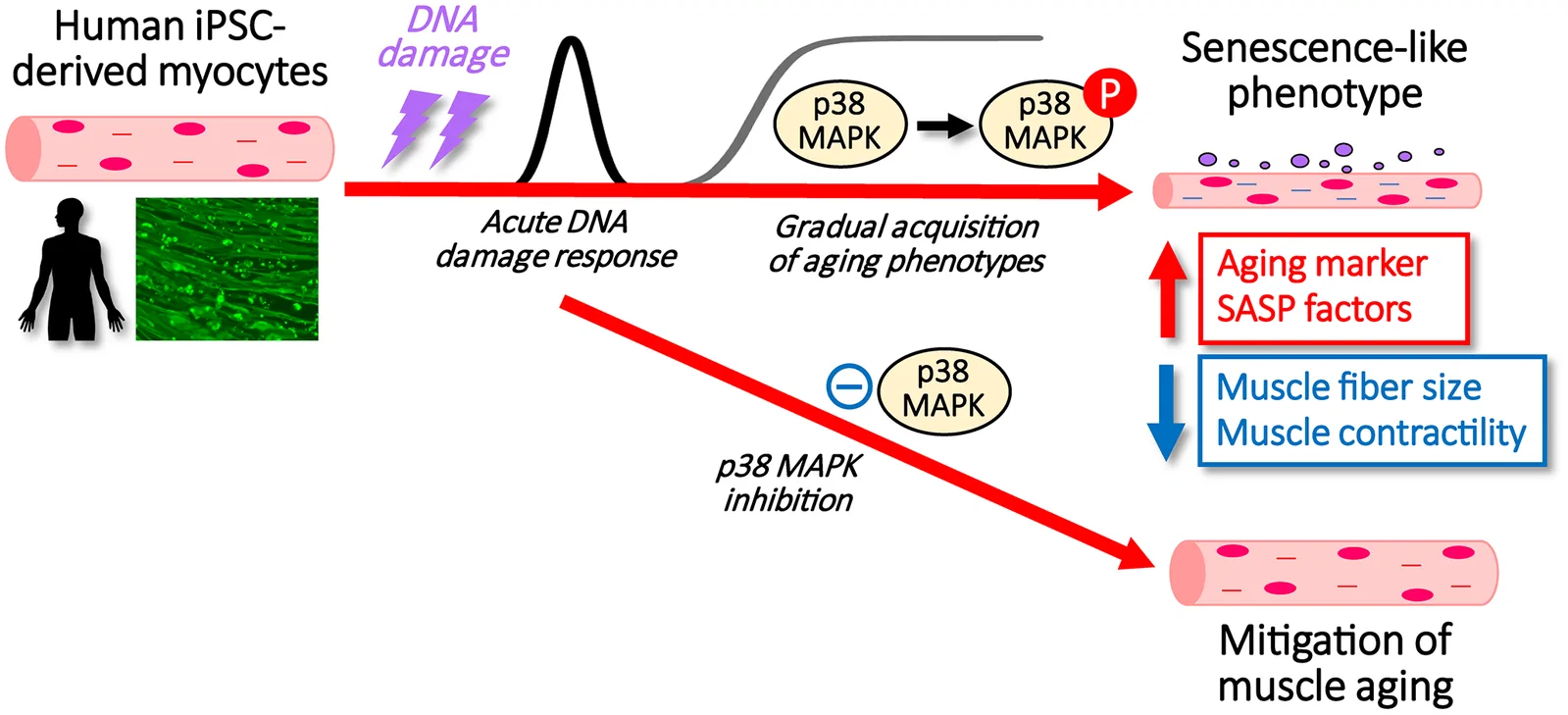
Schematic showing the process after DNA damage to hiPSC-derived myocytes with/without p38MAPK inhibitors.

## MATERIALS AND METHODS

### Materials

Doxorubicin, anisomycin, SB203580, SB202190, and Y-27632 were purchased from FUJIFILM Wako; Arsenite from Nacalai; AZD0530 and GSK2256098 from Cayman; and doxycycline from KLT Laboratories. Anti-lamin B1 and anti-CYR61 antibodies were purchased from ProteinTech; anti-FN1 antibody from BD; and anti-p21, anti-p16, anti-α-tubulin, anti-γH2AX, and p38MAPK antibodies were obtained from Cell Signaling Technology. Dasatinib and anti-β-actin antibody were from Sigma, and anti-myosin heavy chain (MHC) antibody was from R&D systems.

### Cell culture

The human iPS cell (hiPSC) line 409B2^tet-MyoD^ was maintained in StemFit AK02N medium (Ajinomoto) and differentiated into myocytes as previously described [14]. Briefly, on day 0, hiPSCs were seeded on Matrigel-coated plates and cultured in StemFit medium supplemented with 10 μM Y-27632. On day 1, the medium was replaced with Primate ES Cell Medium (Reprocell) containing 10 μM Y-27632 and 3.3 μM all-trans retinoic acid (ATRA). On day 2, cells were treated with Primate ES Cell Medium supplemented with 3.3 μM ATRA and 1 μg/mL doxycycline (Dox) to induce MyoD1 expression. On day 3, the medium was changed to αMEM containing 5% KnockOut Serum Replacement (Gibco), 3.3 μM ATRA and 1 μg/mL Dox, and then cells were incubated for 2–3 days. After differentiation, hiPSC-myocytes were either exposed to X-ray irradiation or treated with doxorubicin. X-ray treatment was performed as previously described [34].

### RNA extraction and quantitative reverse transcription (RT)-PCR

Total RNA was extracted using ISOGEN (NIPPON GENE) according to the manufacturer’s instructions. Complementary DNA (cDNA) was synthesized and amplified using the High-Capacity cDNA Reverse Transcription Kit (Applied Biosystems). Quantitative real-time PCR was performed using an Applied Biosystems StepOnePlus system. Relative mRNA levels were normalized to 18S ribosomal RNA. The primers used for quantitative PCR analysis are listed in Supplementary Table 1.

### Western blot analysis

hiPSC-myocytes were treated as described in the figure legends. Following treatment, cells and media were harvested, and Western blot analysis was conducted as previously described [35].

### Immunofluorescence staining and image analyses

hiPSC-derived myocytes were fixed with 4% paraformaldehyde for 15 min, permeabilized with 0.1% Triton X-100 in PBS for 5 min, and blocked with 3% BSA in PBS for 1 h. Cells were sequentially incubated with an anti-MHC antibody followed by Alexa Fluor 488-conjugated secondary antibodies. Nuclei were counterstained with DAPI (Sigma-Aldrich). Fluorescence images were captured using a BZ-X810 fluorescence microscope (KEYENCE). For quantification of myocyte diameters and MHC-positive areas, images were acquired using the same microscope and analyzed with BZ-X800 Analyzer software (KEYENCE).

### Measurement of contractile activity

To apply electrical pulse stimulation (EPS) to myotubes, hiPSCs were differentiated into myotubes as previously described [14]. EPS conditions followed established protocols [15]. Briefly, from day 7 to day 14 of differentiation, cells were stimulated with electrical pulses at 23 V pulse strength, 4 ms pulse width, and 1 Hz frequency. Pulses were delivered using a C-Dish electrical stimulation chamber and a C-Pace 100 pulse generator (IonOptix). Motion analysis of hiPSC-derived myotubes was performed as described previously [15]. Myotube movements were recorded at 15 frames/s for 25 s using the BZ-X810 fluorescence microscope (KEYENCE). To estimate contractile displacement, three myotubes showing the highest activity were selected from each of three fields in three independent dishes. Displacement was measured using VW-9000 motion analysis software (KEYENCE).

### Statistical analysis

All data are presented as mean ± SD. Statistical analyses were performed using one-way ANOVA followed by Tukey’s post-hoc test or Dunnett’s test. A p-value of <0.05 was considered statistically significant.

## Supplementary Material

Supplementary Figures

Supplementary Table 1

Supplementary Table 2

Supplementary Table 3
