# Supplementary material for: p38MAP kinase regulates senescence in human iPS-derived myocytes
Source: Aging (Albany NY). 2026 May 28;18(1):622–38. doi: 10.18632/aging.206385 (PMC13632860; doi:10.18632/aging.206385)
Supplement: Supplementary file 1 [file aging-18-1-206385-s001.pdf]

SUPPLEMENTARY FIGURES

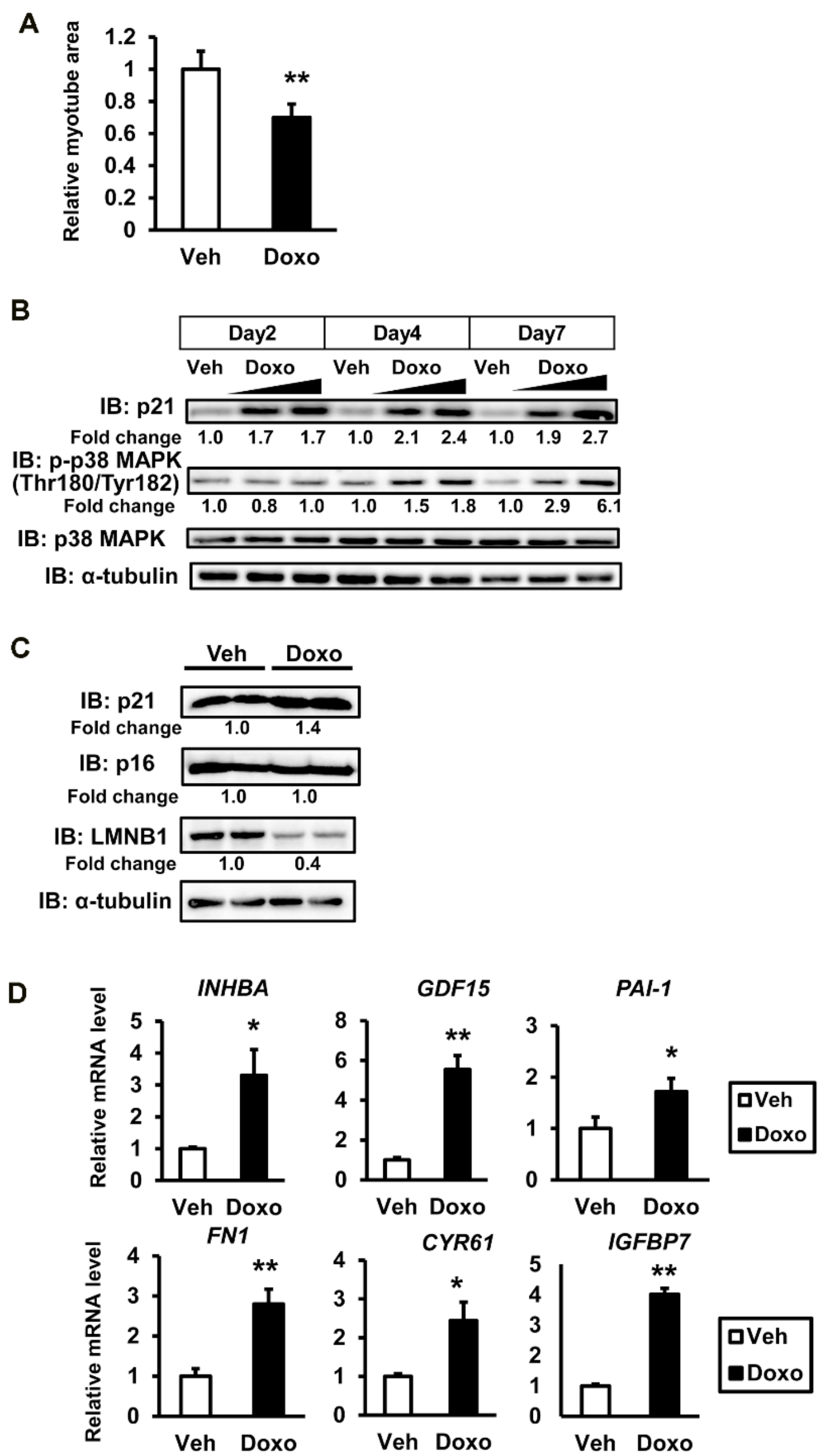

Supplementary Figure 1. Doxorubicin induces senescence in hiPSC-derived myocytes. (**A**) hiPSC-derived myocytes were treated with doxorubicin (doxo, 50 nM). One week after treatment, cells were fixed, permeabilized, and stained for MHC and nuclei. Relative MHC-positive areas were quantified as described in the methods section. The relative myotube area in vehicle-treated controls (Veh) was set to 1. Data are presented as mean ± SD (n = 9), and statistical analysis was performed using Student's t-test. **p < 0.01. (**B**) One week after doxorubicin treatment (doxo, 20 or 50 nM), whole-cell lysates were prepared from hiPSC-derived myocytes and subjected to immunoblotting to analyze the expression of the indicated proteins. (**C**) One week after doxorubicin treatment (doxo, 50 nM), whole-cell lysates were prepared from hiPSC-derived myocytes and subjected to immunoblotting to analyze the expression of the indicated proteins. (**D**) Representative mRNA expression data from hiPSC-derived myocytes treated with doxorubicin (doxo, 50 nM). One week after treatment, total RNA was isolated and analyzed by real-time PCR. Expression levels were normalized to 18S rRNA and presented as relative values. Data are presented as mean ± SD (n = 3), and statistical analysis was performed using Student's t-test. **p < 0.01. The numbers under the lanes indicate fold changes in protein level relative to the control and were standardized against total p38MAPK or α-tubulin.

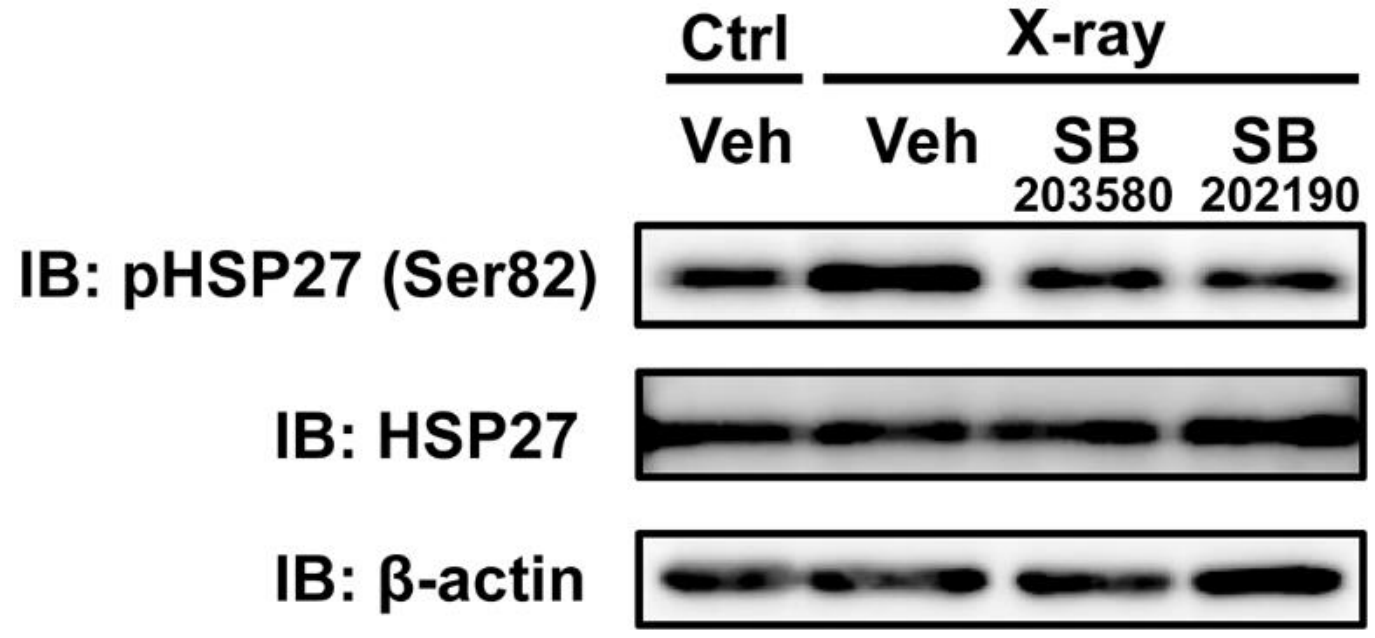

Supplementary Figure 2. Phosphorylation of HSP27 is suppressed by p38MAPK inhibitors. Whole-cell lysates from hiPSC-derived myocytes were collected following X-ray irradiation. Lysates were subjected to immunoblotting to assess the expression and phosphorylation status of the indicated proteins.

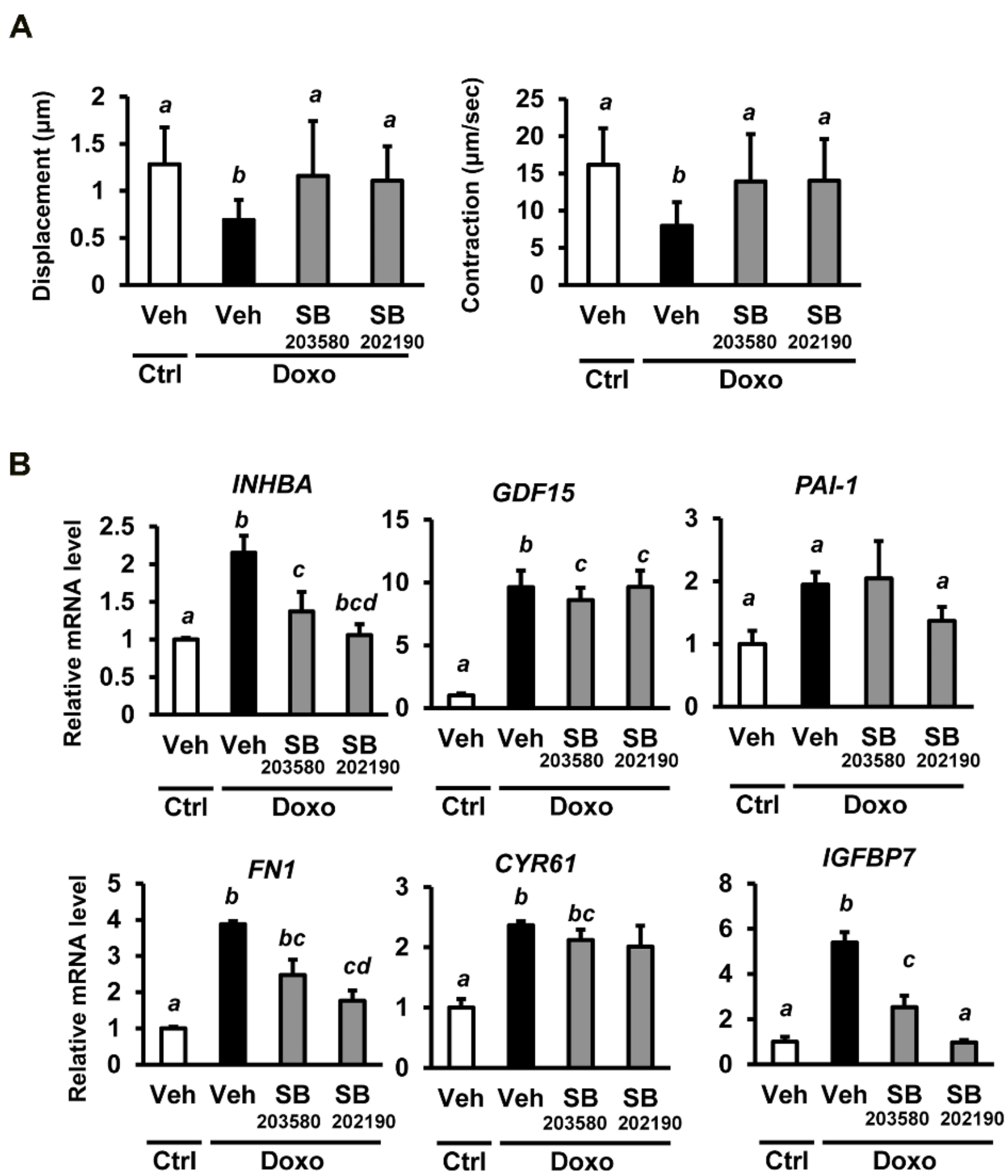

Supplementary Figure 3. Doxorubicin-induced senescence in hiPSC-derived myocytes is mediated via the p38MAPK pathway. (**A**) hiPSC-derived myocytes were treated with doxorubicin (doxo, 50 nM). Twenty-four hours later, cells were treated with p38MAPK inhibitors (SB203580 or SB202190). Myocytes were then stimulated with electrical pulses (23 V, 4 ms, 1 Hz) and myotube movement was quantified using motion analysis software. (**B**) One week after doxorubicin treatment, total RNA was extracted and analyzed by real-time PCR. mRNA levels were normalized to 18S rRNA, and expression in untreated controls (Veh) was set to 1. Data are presented as mean ± SD (n = 3 or 27). Statistical significance was determined using Tukey's test. Different letters indicate significant differences between groups ($p < 0.05$).

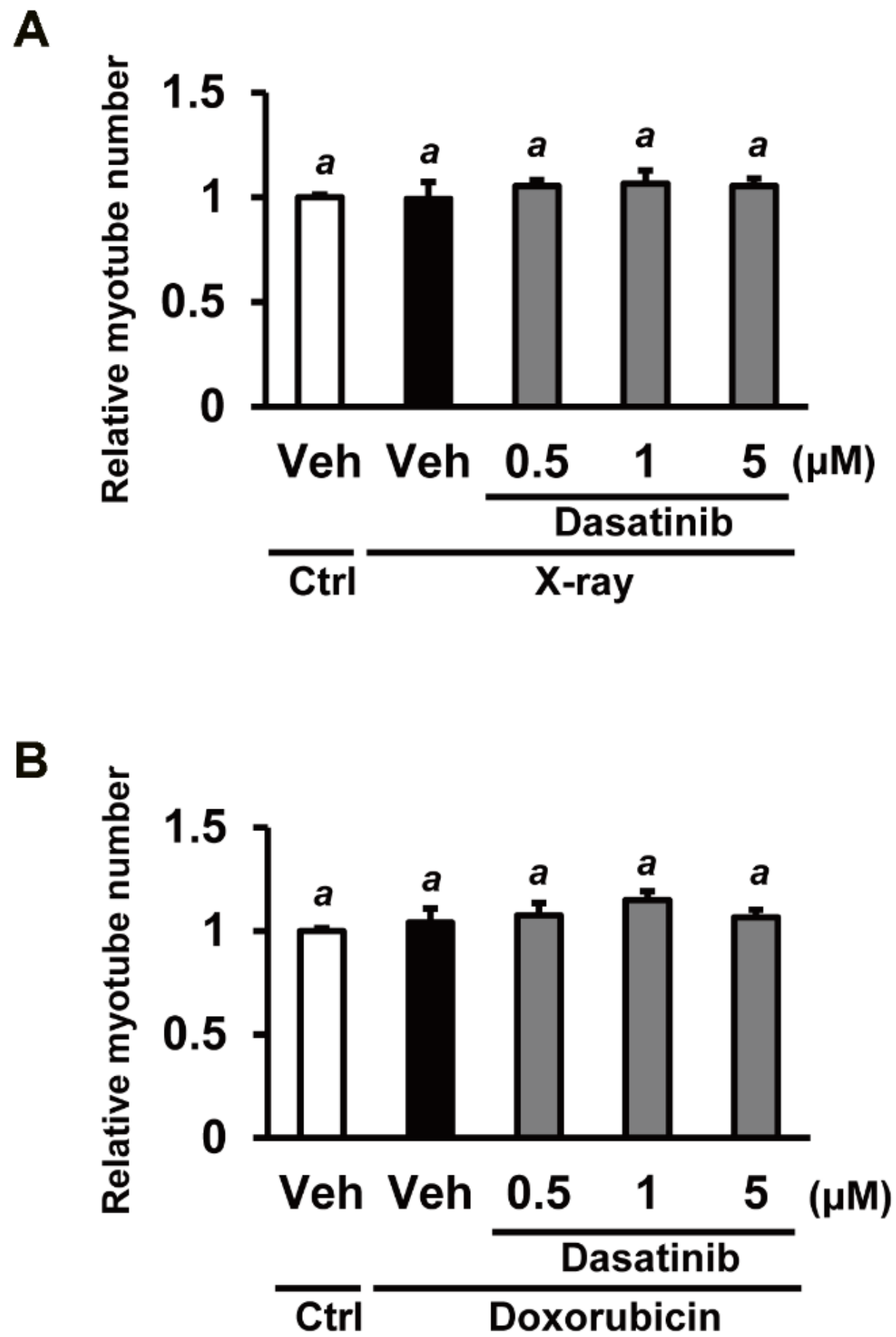

Supplementary Figure 4. Myotube numbers are not affected by senescence and dasatinib in hiPSC-derived myocytes. One week after X-ray irradiation (**A**) or doxorubicin treatment (**B**) together with dasatinib treatment, cells were fixed, permeabilized, and stained for MHC. The number of MHC-positive myotubes in each field were counted. The relative myotube numbers in vehicles without X-ray irradiation or doxorubicin were set to 1. Data are presented as mean ± SE (n = 5), and statistical analysis was performed using Tukey's test. Different letters indicate significant differences between groups ($p < 0.05$).

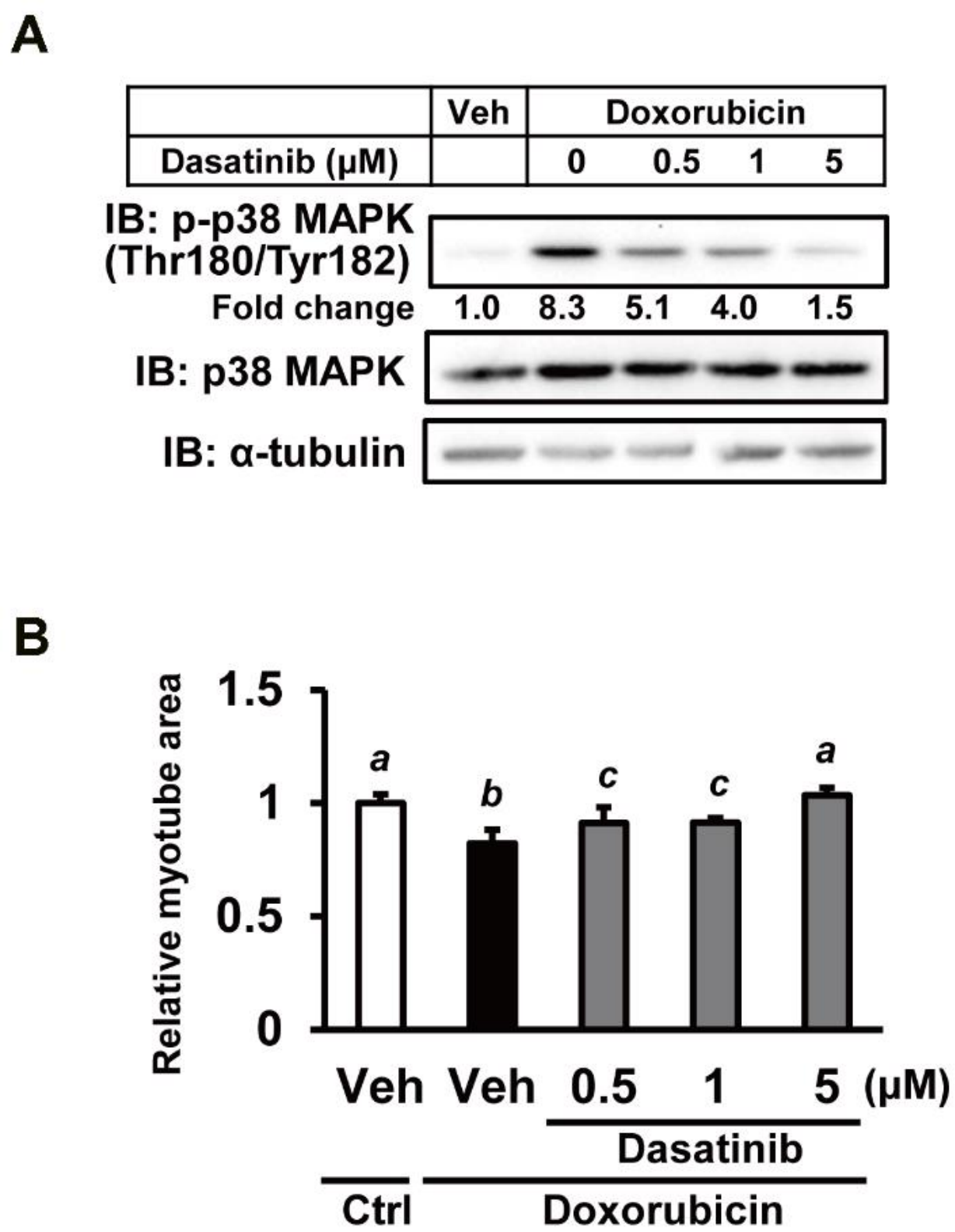

Supplementary Figure 5. Dasatinib reduces p38MAPK activation and myofiber atrophy by doxorubicin in hiPSC-derived myocytes. (**A**) hiPSC-derived myocytes were treated with doxorubicin (doxo, 50 nM). One week after treatment, cells were then treated with dasatinib for 6hrs. Whole-cell lysates were prepared from hiPSC-derived myocytes and subjected to immunoblotting to analyze the expression of the indicated proteins. The numbers under the lanes indicate fold changes in protein level relative to the control and were standardized against total p38MAPK. (**B**) hiPSC-derived myocytes were treated with doxorubicin (doxo, 50 nM) and dasatinib. One week after treatment, cells were fixed, permeabilized, and stained for MHC and nuclei. Relative MHC-positive areas were quantified as described in the methods section. The relative myotube area in controls (Ctrl) without both doxorubicin and dasatinib was set to 1. Data are presented as mean ± SD (n = 9), and statistical significance was determined using Tukey's test. Different letters indicate significant differences between groups ($p < 0.05$).
