# Supplementary material for: p38MAP kinase regulates senescence in human iPS-derived myocytes
Source: Aging (Albany NY). 2026 May 28;18(1):622–38. doi: 10.18632/aging.206385 (PMC13632860; doi:10.18632/aging.206385)
Supplement: Supplementary file 2 [file aging-18-1-206385-s002.pdf]

SUPPLEMENTARY TABLES

Supplementary Table 1. List of primer sequences used in real-time PCR.

Gene	Forward primer 5′-3′	Reverse primer 5′-3′
18S	ACCGCAGCTAGGAATAATGGA	GCCTCAGTTCCGAAAACCA
p21	TGGAGACTCTCAGGGTCGAAA	GGCGTTTGGAGTGGTAGAAATC
p16	AGCTGTCGACTTCATGACAAG	GAGCTTTGGTTCTGCCATTTG
LMNB1	AAGCAGCTGGAGTGGTTGTT	TTGGATGCTCTTGGGGTTC
INHBA	TCTGCAGTAGTGTGGACTAGAA	CCTGGGTAATTGGGTAGGAAAG
GDF15	CTACAATCCCATGGTGCTCAT	TCATATGCAGTGGCAGTCTTT
PAI-1	CATTACTACGACATCCTGGAACTG	AATGTTGGTGAGGGCAGAGAG
IGFBP7	GGGTGCTGGTATCTCCTCTA	TGTAAGGCATCAACCACTGTAA
FN1	CTGAGACCACCATCACCATTAG	GATGGTTCTCTGGATTGGAGTC
CYR61	CAGGACTGTGAAGATGCGGT	GCCTGTAGAAGGGAAAGGT
CTGF	AGGAGTGGGTGTGTGACGA	CCAGGCAGTTGGCTCTAATC
ITGA2	CTGTTCAAGGAGGAGACAACTT	CTGCACCCAGCATCAGAATA
ITGA4	CCAGCAGAGAAGCTAACTGTAG	CATGAGGACCAAGGTGGTAAG
ITGA5	GGTGGACCAGGAAGCTATTT	GAACCAGGTTGATCAGGTACTC
ITGA6	CCAAGGTTCTGAGCCCAAATA	GGGAATGGGACGCAGTTTAT
ITGA7	CCTTAGAGTGCTGTGAGATGAG	GAATGGGAGAGGAAGGGATTAG
ITGA11	CCTACAGCACGGTCCTAAATATC	CTCCTCGTTCACACACTCAAT
ITGAV	ACTCTTAGCTGGTCTTCGTTTC	TGTGAGATACAACTGGGCTTAC
ITGB1	CCCAGAGGCTCCAAAGATATAAA	GCTGTGGTTGGATCTGAGTAA
ITGB3	CATCCATAGCACCTCCACATAC	CCAGCCAACTCATGGGAATAA
ITGB5	CAGATGACACCACAGGAGATTG	GTAGTACAGGTCCACAGGATAGT
ITGB8	GGCCAAGGTGAAGACAATAGA	ATCCTCTTGAACACACCATCC
