# Supplementary material for: p38MAP kinase regulates senescence in human iPS-derived myocytes
Source: Aging (Albany NY). 2026 May 28;18(1):622–38. doi: 10.18632/aging.206385 (PMC13632860; doi:10.18632/aging.206385)
Supplement: Supplementary file 3 [file aging-18-1-206385-s003.docx]

Supplementary Table 2. List of 65 proteins increased by X-ray irradiation in proteome analysis.

| Protein name | Fold change (>1.5, X-ray/Ctrl) | | |
| --- | --- | --- | --- |
|  | Day 2 | Day 4 | Day 7 |
| ANOS1 | 1.029854819 | 2.760930174 | 9.565046252 |
| CRHBP | 0.66237468 | 4.335077357 | 6.961702023 |
| ADAMTS5 | 0.742179286 | 1.009468477 | 6.792844818 |
| GDF6 | N.D. | 1.985812764 | 6.400009978 |
| PAI1 | 1.577331889 | 4.728568454 | 6.276523343 |
| ADAMTS15 | 0.987904759 | 1.667064518 | 6.006877694 |
| S100A9 | N.D. | N.D. | 5.113968096 |
| GDF15 | 2.16677908 | 4.013244839 | 4.74590632 |
| LOXL4 | 1.193362633 | 2.771403368 | 4.328426601 |
| CCDC3 | 1.222446466 | 1.468467551 | 4.111545017 |
| NPPB | 1.078003521 | 2.115435572 | 3.996257163 |
| LEFTY1 | N.D. | 0.97216956 | 3.649033062 |
| PRADC1 | 0.314665783 | 1.106502333 | 3.272137961 |
| CILP | N.D. | 1.761012269 | 3.187881549 |
| HTRA1 | 0.486977429 | 0.979611081 | 3.005905611 |
| LEFTY2 | 0.418349265 | 1.592828241 | 3.00085754 |
| IL17B | 0.588605233 | 0.550988548 | 2.991736785 |
| IGFBP2 | 2.981640053 | 3.233087408 | 2.918607201 |
| TSKU | 1.155288484 | 0.362044157 | 2.730367077 |
| SEMA3G | 2.194214841 | 1.88519665 | 2.70951828 |
| DKK2 | N.D. | N.D. | 2.686491378 |
| FGG | 2.081030862 | 1.294739699 | 2.532109605 |
| MATN2 | 0.777950512 | 1.59617054 | 2.509857779 |
| MXRA7 | 1.436917037 | 1.032366386 | 2.508864518 |
| SFN | 0.74064279 | 1.788693503 | 2.495894272 |
| S100A8 | N.D. | N.D. | 2.456704128 |
| DCD | N.D. | N.D. | 2.435386922 |
| NT5DC3 | 0.352865485 | 1.497070989 | 2.38208519 |
| FGF1 | 1.251532132 | 2.097253138 | 2.380373321 |
| SPATA20 | 0.537480599 | 0.784613594 | 2.316353522 |
| PLAU | 1.40617563 | 2.011008564 | 2.273539256 |
| SMPD1 | 0.686158282 | 1.403641551 | 2.267230318 |
| CPA4 | 2.620703208 | 1.517216902 | 2.242778667 |
| PAPPA | 1.068669981 | 3.386714733 | 2.239900596 |
| GAS6 | N.D. | N.D. | 2.197707763 |
| FSTL3 | 2.73878602 | 2.2724133 | 2.197412624 |
| INHBA | 2.275823654 | 1.443741365 | 2.196968948 |
| EDN1 | 1.916556877 | 2.970976651 | 2.092600812 |
| HGFAC | N.D. | N.D. | 2.061698481 |
| TRH | 0.183804149 | 0.347630556 | 2.050537745 |
| FN1 | 0.494327656 | 1.286030349 | 2.009123682 |
| LGI4 | 0.521113465 | 0.850936067 | 2.007492922 |
| SEMA3F | 0.939551865 | 1.639600671 | 2.000033727 |
| TNC | 0.976449624 | 1.411520385 | 1.894433697 |
| WNT5A | 0.816120847 | 0.999124444 | 1.884717143 |
| S100A7 | N.D. | N.D. | 1.82886734 |
| RSPRY1 | 0.753321325 | 1.131374433 | 1.812653835 |
| LYZ | N.D. | N.D. | 1.799685012 |
| MATN3 | 0.219930904 | 0.935266958 | 1.798205915 |
| VASH2 | N.D. | 2.443630383 | 1.785232249 |
| ALB | 1.103891729 | 2.094242456 | 1.769962583 |
| CYR61 | 1.672019366 | 1.856346158 | 1.767026352 |
| IGFBP3 | 0.685181239 | 0.78190103 | 1.758623013 |
| SRPX | 0.383732866 | 1.344118632 | 1.752668197 |
| EDIL3 | 1.788315543 | 0.964329761 | 1.678636136 |
| TIMP3 | 0.887774633 | 0.943447375 | 1.665577931 |
| GATD1 | 0.85474715 | 1.448426575 | 1.61647684 |
| CCDC134 | 0.689209786 | 1.537187919 | 1.610287159 |
| CLU | 0.751909178 | 1.17194101 | 1.589039605 |
| NTN3 | 1.606627525 | 0.817897225 | 1.550737111 |
| RGMA | 4.264600722 | 1.386754906 | 1.538102337 |
| NOG | 0.842426778 | 0.969468497 | 1.518048416 |
| SEMA3C | 1.123641122 | 0.812776784 | 1.517996794 |
| SRPX2 | 1.057061761 | 1.737073712 | 1.517191297 |
| FAM177A1 | 0.947674645 | 0.840676434 | 1.514409758 |
| ANOS1 | 1.029854819 | 2.760930174 | 9.565046252 |
| CRHBP | 0.66237468 | 4.335077357 | 6.961702023 |
| ADAMTS5 | 0.742179286 | 1.009468477 | 6.792844818 |
| GDF6 | N.D. | 1.985812764 | 6.400009978 |
| PAI1 | 1.577331889 | 4.728568454 | 6.276523343 |
| ADAMTS15 | 0.987904759 | 1.667064518 | 6.006877694 |
| S100A9 | N.D. | N.D. | 5.113968096 |
| GDF15 | 2.16677908 | 4.013244839 | 4.74590632 |
| LOXL4 | 1.193362633 | 2.771403368 | 4.328426601 |
| CCDC3 | 1.222446466 | 1.468467551 | 4.111545017 |
| NPPB | 1.078003521 | 2.115435572 | 3.996257163 |
| LEFTY1 | N.D. | 0.97216956 | 3.649033062 |
| PRADC1 | 0.314665783 | 1.106502333 | 3.272137961 |
| CILP | N.D. | 1.761012269 | 3.187881549 |
| HTRA1 | 0.486977429 | 0.979611081 | 3.005905611 |
| LEFTY2 | 0.418349265 | 1.592828241 | 3.00085754 |
| IL17B | 0.588605233 | 0.550988548 | 2.991736785 |
| CCN2 | 2.981640053 | 3.233087408 | 2.918607201 |
| TSKU | 1.155288484 | 0.362044157 | 2.730367077 |
| SEMA3G | 2.194214841 | 1.88519665 | 2.70951828 |
| DKK2 | N.D. | N.D. | 2.686491378 |
| FGG | 2.081030862 | 1.294739699 | 2.532109605 |
| MATN2 | 0.777950512 | 1.59617054 | 2.509857779 |
| MXRA7 | 1.436917037 | 1.032366386 | 2.508864518 |
| SFN | 0.74064279 | 1.788693503 | 2.495894272 |
| S100A8 | N.D. | N.D. | 2.456704128 |
| DCD | N.D. | N.D. | 2.435386922 |
| NT5DC3 | 0.352865485 | 1.497070989 | 2.38208519 |
| FGF1 | 1.251532132 | 2.097253138 | 2.380373321 |
| SPATA20 | 0.537480599 | 0.784613594 | 2.316353522 |
| PLAU | 1.40617563 | 2.011008564 | 2.273539256 |
| SMPD1 | 0.686158282 | 1.403641551 | 2.267230318 |
| CPA4 | 2.620703208 | 1.517216902 | 2.242778667 |
| PAPPA | 1.068669981 | 3.386714733 | 2.239900596 |
| GAS6 | N.D. | N.D. | 2.197707763 |
| FSTL3 | 2.73878602 | 2.2724133 | 2.197412624 |
| INHBA | 2.275823654 | 1.443741365 | 2.196968948 |
| EDN1 | 1.916556877 | 2.970976651 | 2.092600812 |
| HGFAC | N.D. | N.D. | 2.061698481 |
| TRH | 0.183804149 | 0.347630556 | 2.050537745 |
| FN1 | 0.494327656 | 1.286030349 | 2.009123682 |
| LGI4 | 0.521113465 | 0.850936067 | 2.007492922 |
| SEMA3F | 0.939551865 | 1.639600671 | 2.000033727 |
| TNC | 0.976449624 | 1.411520385 | 1.894433697 |
| WNT5A | 0.816120847 | 0.999124444 | 1.884717143 |
| S100A7 | N.D. | N.D. | 1.82886734 |
| RSPRY1 | 0.753321325 | 1.131374433 | 1.812653835 |
| LYZ | N.D. | N.D. | 1.799685012 |
| MATN3 | 0.219930904 | 0.935266958 | 1.798205915 |
| VASH2 | N.D. | 2.443630383 | 1.785232249 |
| ALB | 1.103891729 | 2.094242456 | 1.769962583 |
| CYR61 | 1.672019366 | 1.856346158 | 1.767026352 |
| IGFBP3 | 0.685181239 | 0.78190103 | 1.758623013 |
| SRPX | 0.383732866 | 1.344118632 | 1.752668197 |
| EDIL3 | 1.788315543 | 0.964329761 | 1.678636136 |
| TIMP3 | 0.887774633 | 0.943447375 | 1.665577931 |
| GATD1 | 0.85474715 | 1.448426575 | 1.61647684 |
| CCDC134 | 0.689209786 | 1.537187919 | 1.610287159 |
| CLU | 0.751909178 | 1.17194101 | 1.589039605 |
| NTN3 | 1.606627525 | 0.817897225 | 1.550737111 |
| RGMA | 4.264600722 | 1.386754906 | 1.538102337 |
| NOG | 0.842426778 | 0.969468497 | 1.518048416 |
| SEMA3C | 1.123641122 | 0.812776784 | 1.517996794 |
| SRPX2 | 1.057061761 | 1.737073712 | 1.517191297 |
| FAM177A1 | 0.947674645 | 0.840676434 | 1.514409758 |

The data were presented as fold changes in protein level relative to the control. N.D.: Not detected.
