# Supplementary material for: p38MAP kinase regulates senescence in human iPS-derived myocytes
Source: Aging (Albany NY). 2026 May 28;18(1):622–38. doi: 10.18632/aging.206385 (PMC13632860; doi:10.18632/aging.206385)
Supplement: Supplementary file 4 [file aging-18-1-206385-s004.docx]

Supplementary Table 3. Gene list from RNA sequencing analysis of X-ray irradiation and p38MAPK inhibitor experiments.

|  | Fold change | Gene list | |
| --- | --- | --- | --- |
| Gene name | Xray/Ctrl > 2 | Xray/Ctrl > 2, and  Xray/SB202190 < 0.66 | Xray/Ctrl > 2, and  Xray/SB203580 < 0.66 |
| MIR5187 | 11047.1 |  |  |
| MIR7855 | 4374 | * |  |
| MIR943 | 1130.552273 | * | * |
| LINC01561 | 961.712766 | * | * |
| MIR9898 | 853.2574526 |  | * |
| MIR4658 | 294.1949434 |  |  |
| RPL39P37 | 286.1463023 | * | * |
| TOMM20L | 238.2452055 |  |  |
| C21orf91-OT1 | 149.5363985 | * |  |
| MIR631 | 146.2353036 |  | * |
| HNRNPA1P63 | 105.3943746 |  | * |
| LOC101927345 | 88.85454851 | * |  |
| MIR1909 | 88 | * |  |
| SNORD160 | 83.91866649 | * |  |
| MIR1287 | 75.919108 | * | * |
| MIR6784 | 71 |  |  |
| MIR450A1 | 68.05538263 |  |  |
| PPIAP30 | 54.46292071 |  |  |
| MIR3652 | 53.9729973 | * | * |
| MIR761 | 53.55608028 | * | * |
| PPIAP13 | 47.8864084 |  | * |
| LHX4-AS1 | 41.63979054 | * | * |
| MIR6852 | 34.3383345 |  | * |
| PPIAP43 | 32.47505706 |  |  |
| MIR2110 | 30.21533442 | * | * |
| FOXC2-AS1 | 27.3601434 |  |  |
| MIR4721 | 27.07299435 |  | * |
| TRGC2 | 25.72197879 | * | * |
| LOC112268055 | 22.76878307 | * |  |
| MIR10392 | 21.30526316 | * | * |
| SNORA9B | 20.74191108 |  |  |
| MIR658 | 20.6010101 | * | * |
| MIR762 | 20.30927647 | * | * |
| LOC642677 | 19.91105769 |  | * |
| LOC105375785 | 19.47024609 | * | * |
| PZP | 18.88078292 | * | * |
| RAET1E-AS1 | 18.56453559 |  |  |
| SPATA21 | 18.48305085 | * |  |
| CST1 | 18.3361267 | * | * |
| FLJ38576 | 18.07437132 | * | * |
| RPL9P3 | 16.76251633 | * | * |
| LOC112268323 | 16.09931707 |  | * |
| EDN2 | 15.98504638 |  | * |
| CDK2AP2P3 | 15.64544184 | * |  |
| DOCK9-DT | 15.41938998 |  |  |
| SETP20 | 15.40321058 |  | * |
| ARMS2 | 15.37880219 | * |  |
| SELENOKP1 | 15.34336203 |  |  |
| PFN1P2 | 14.97706322 |  |  |
| FGF19 | 14.79092715 | * | * |
| SNORD127 | 13.52760795 |  |  |
| AMY1B | 13.4599636 | * | * |
| LOC105370733 | 12.96771424 |  |  |
| HTR1A | 12.85727063 | * |  |
| TUBB8P11 | 12.82675602 | * | * |
| RPS29P18 | 12.23041623 | * |  |
| LOC105377329 | 12.09014253 |  |  |
| MIR3657 | 12.06694232 |  |  |
| LIVAR | 11.90291019 |  | * |
| MIR639 | 11.84781722 | * | * |
| SPRR2G | 11.40133885 |  |  |
| SNORD1B | 11.22037006 | * |  |
| MIR4649 | 11.16597473 | * | * |
| HNRNPA1P26 | 10.7290566 |  | * |
| LINC02075 | 10.53526618 | * | * |
| LOC105369592 | 10.41032038 | * | * |
| PLA2G10 | 10.34516702 | * | * |
| SNORD50A | 10.33536789 | * | * |
| LOC105377650 | 10.32688479 | * | * |
| STIP1P3 | 10.31917544 |  |  |
| LINC02773 | 10.29452725 | * | * |
| MIR612 | 10.26415094 |  | * |
| RPL15P8 | 10.19103522 |  |  |
| MIR155HG | 9.993611856 |  |  |
| MIR12121 | 9.888888889 | * | * |
| CPA4 | 9.870912034 | * | * |
| FGF3 | 9.70487622 |  | * |
| SLCO2B1 | 9.679222778 |  | * |
| NPPB | 9.656326173 | * | * |
| NHLH2 | 9.412554705 |  | * |
| LOC105374308 | 9.30366568 | * |  |
| LINC02697 | 9.300858613 | * | * |
| TRH-GTG1-8 | 9.165914446 | * | * |
| LOC105372470 | 9.154368008 |  |  |
| NBPF4 | 9.121410226 |  |  |
| HERC2P1 | 9.072400084 | * |  |
| SNORD121B | 9.026954178 |  |  |
| RPL31P17 | 8.978305286 |  | * |
| RPL32P18 | 8.934115226 | * | * |
| LOC105371976 | 8.908092793 | * | * |
| RPL39P15 | 8.867513268 |  |  |
| KRT7 | 8.8322305 | * | * |
| RPL21P46 | 8.600343803 | * |  |
| GRK7 | 8.599624354 | * | * |
| EPHA8 | 8.583739567 | * |  |
| SPOCD1 | 8.365874483 |  |  |
| RPS29P16 | 8.202985527 | * | * |
| LOC112268133 | 8.200136729 |  |  |
| SNORD116-5 | 8.154538247 |  | * |
| ACSL5 | 8.147567977 |  | * |
| MIR3132 | 8.11411855 |  |  |
| B4GALT1-AS1 | 8.093846665 | * |  |
| IVL | 8.076592956 | * | * |
| EDN1 | 8.050265303 | * |  |
| TREX1 | 8.02763738 |  |  |
| NECTIN4 | 7.974721921 | * | * |
| SNX22 | 7.935818105 | * | * |
| NECTIN4-AS1 | 7.860406506 |  | * |
| LOC105371908 | 7.805403723 |  | * |
| LOC105378076 | 7.772820396 |  |  |
| CEP295NL | 7.759246834 | * | * |
| CXCL5 | 7.711904012 | * | * |
| LOC105375242 | 7.627662011 |  |  |
| PLPP4 | 7.568495352 |  |  |
| C10orf62 | 7.56679695 |  | * |
| MIR5093 | 7.562793668 | * | * |
| LOC105371409 | 7.524326966 |  |  |
| PIN4P1 | 7.482693386 | * | * |
| LIMS3-LOC440895 | 7.417105354 |  | * |
| LOC105375426 | 7.360236968 |  | * |
| STYK1 | 7.336990971 | * | * |
| TLR1 | 7.333094992 |  | * |
| ISCA1P1 | 7.242591927 |  | * |
| IL11 | 7.20074102 |  |  |
| FLJ31104 | 7.196880338 | * | * |
| COLEC10 | 7.195875685 | * | * |
| TP53AIP1 | 7.176673179 |  |  |
| LINC00515 | 7.160227477 | * |  |
| LOC112268444 | 7.125196713 | * |  |
| IL33 | 7.101277062 |  |  |
| DHRS2 | 6.991258937 |  |  |
| PTK6 | 6.968238549 | * | * |
| RPS10P7 | 6.935151918 |  |  |
| MFNG | 6.934795617 |  |  |
| RAD51L3-RFFL | 6.918050292 |  |  |
| SNRPGP2 | 6.911809689 | * | * |
| SERPINE1 | 6.898981044 |  |  |
| LY86 | 6.892868477 |  |  |
| LOC105372956 | 6.861065282 | * | * |
| LOC105378342 | 6.82178807 |  |  |
| LOC105374798 | 6.795791456 | * | * |
| RN7SKP175 | 6.795462132 | * | * |
| SNORA51 | 6.749666691 |  |  |
| REN | 6.742584222 | * | * |
| LRAT | 6.699384892 | * | * |
| LOC100507472 | 6.688813174 | * |  |
| COL8A1 | 6.640568431 | * | * |
| LINC01357 | 6.629112007 |  | * |
| TRIM67 | 6.575916438 | * | * |
| S100A8 | 6.56576459 | * |  |
| SNORA77B | 6.561085973 | * | * |
| ZSCAN10 | 6.532927885 |  | * |
| LOC112267929 | 6.523495959 | * | * |
| LINC02193 | 6.51921757 |  | * |
| LOC105371910 | 6.464301113 |  | * |
| LOC105377137 | 6.449646162 |  | * |
| MYEOV | 6.446374936 |  | * |
| IL10 | 6.43082965 | * |  |
| MIR6791 | 6.415629809 |  |  |
| LOC102723345 | 6.393173048 | * |  |
| LOC105370767 | 6.390039335 | * | * |
| CRYAA | 6.368628322 | * | * |
| CRYAA2 | 6.36807984 | * | * |
| LOC107987258 | 6.348002509 | * | * |
| LOC105370363 | 6.341988581 |  | * |
| UGT8 | 6.320320623 | * | * |
| RPLP1P1 | 6.305231633 |  |  |
| TAS2R43 | 6.27127525 |  |  |
| LOC107986524 | 6.265196709 | * | * |
| LINC02182 | 6.255938728 |  |  |
| ECEL1P2 | 6.252691474 |  | * |
| WDR87 | 6.251332167 | * | * |
| LOC105372493 | 6.220749627 |  | * |
| NBPF6 | 6.218687207 |  |  |
| GALNT6 | 6.208356289 |  |  |
| LOC105369623 | 6.204112978 |  | * |
| CCER2 | 6.170423263 | * |  |
| LOC105379224 | 6.138560014 |  |  |
| IGLV1-44 | 6.130892017 |  |  |
| WFDC21P | 6.125301124 |  |  |
| OR1E2 | 6.121036971 | * | * |
| ARHGAP40 | 6.113772141 |  |  |
| TNFRSF9 | 6.102802072 | * |  |
| SNORA105A | 6.076107461 | * |  |
| LOC105370707 | 6.061709803 |  |  |
| LINC02869 | 6.060030069 | * | * |
| LOC105374235 | 6.044746075 | * | * |
| WNK4 | 6.030120704 | * | * |
| PLCE1-AS1 | 5.976560319 | * | * |
| RAB27B | 5.959638208 |  |  |
| ACTG1P4 | 5.95177711 |  |  |
| LOC105374248 | 5.923893446 | * |  |
| F2RL1 | 5.892001383 |  |  |
| LOC105376218 | 5.891015898 |  |  |
| LOC105369370 | 5.875130529 |  |  |
| NT5E | 5.838025903 |  |  |
| AANAT | 5.803685757 |  |  |
| LOC105372529 | 5.785898877 |  |  |
| WWC2-AS1 | 5.780391734 | * | * |
| LOC107986124 | 5.756449557 |  | * |
| SNORD83A | 5.754790256 | * | * |
| BHLHE40-AS1 | 5.751131603 |  |  |
| LOC107985634 | 5.736446671 |  |  |
| ACSM5 | 5.734698101 | * | * |
| LOC107985207 | 5.69958871 |  | * |
| COMMD3-BMI1 | 5.697029485 | * |  |
| PTCHD4 | 5.695364218 |  |  |
| HCGVIII-2 | 5.689150355 |  |  |
| MAB21L4 | 5.686801432 | * |  |
| LOC101927013 | 5.659838348 |  |  |
| RAB1AP1 | 5.658632222 | * |  |
| FLJ22447 | 5.654104471 | * |  |
| LOC101928021 | 5.650904864 |  |  |
| PDGFB | 5.638492961 |  |  |
| ADRA1D | 5.627776333 | * |  |
| LOC105375167 | 5.622451891 |  |  |
| CBR3-AS1 | 5.611075962 |  | * |
| TNFSF15 | 5.610956562 |  |  |
| LOC105375120 | 5.605138078 |  |  |
| OXCT2P1 | 5.595744096 |  |  |
| CCL3L3 | 5.574303882 | * | * |
| LOC91370 | 5.568866023 |  | * |
| WNT7B | 5.567414783 | * | * |
| DLX5 | 5.557482548 | * | * |
| ARSI | 5.557003863 |  |  |
| FAM178B | 5.547140367 | * |  |
| CRHBP | 5.546865123 |  |  |
| RAET1E | 5.524161644 | * | * |
| SYTL5 | 5.492627372 | * | * |
| LOC105373968 | 5.459763225 |  | * |
| MIR6506 | 5.457391304 |  | * |
| LOC105371967 | 5.450868026 | * |  |
| PLSCR2 | 5.41527661 | * | * |
| CALML3-AS1 | 5.405101163 |  |  |
| S100A9 | 5.398255135 | * | * |
| EEF1E1-BLOC1S5 | 5.396685139 | * | * |
| LOC105374639 | 5.395552094 | * | * |
| IL10RA | 5.379593669 | * | * |
| LINC00312 | 5.373134328 |  |  |
| LOC105373018 | 5.364100135 |  |  |
| TOGARAM2 | 5.349133535 | * | * |
| LINC01749 | 5.346240113 |  |  |
| RNU6-7 | 5.344223195 | * | * |
| CYP24A1 | 5.326333447 | * | * |
| DIRC3-AS1 | 5.285900432 | * | * |
| IFI30 | 5.282628458 |  |  |
| PURPL | 5.268969046 |  |  |
| HSD17B3 | 5.268614625 | * |  |
| LOC389641 | 5.25617067 |  | * |
| PLAC8 | 5.250226678 | * | * |
| LOC107987300 | 5.229066565 |  |  |
| SSTR5-AS1 | 5.225030158 | * | * |
| LOC105376453 | 5.206206388 | * | * |
| LINC02018 | 5.185177984 | * | * |
| LOC105378626 | 5.182577388 |  | * |
| UTS2B | 5.174970834 |  | * |
| LOC105378585 | 5.148431297 | * | * |
| SNORD21 | 5.139507944 | * | * |
| TNFRSF10C | 5.127941119 |  |  |
| RHOH | 5.123333333 |  |  |
| LOC151760 | 5.121309731 | * | * |
| RPL36AP26 | 5.117671502 | * | * |
| LOC112267859 | 5.109301104 | * |  |
| LINC02809 | 5.101843661 |  | * |
| TSBP1-AS1 | 5.100489392 | * |  |
| HS3ST5 | 5.100437777 | * | * |
| LOC105374299 | 5.092661147 |  |  |
| LOC105372710 | 5.091360781 |  | * |
| LOC102724521 | 5.084695156 |  | * |
| H3C14 | 5.078960419 |  |  |
| LOC105371855 | 5.06307021 | * |  |
| NUFIP1P1 | 5.04962708 | * | * |
| GBP3 | 5.036224283 |  |  |
| MYL10 | 5.028361413 |  | * |
| CDC27P11 | 5.02583423 |  |  |
| GATA4 | 5.019674355 | * | * |
| MIR22 | 5.000634518 |  | * |
| TNFRSF6B | 4.992545002 |  |  |
| LOC105371712 | 4.985082517 |  | * |
| HNRNPA1P71 | 4.962058126 |  |  |
| CHCHD2P2 | 4.956701732 | * |  |
| ICAM4 | 4.92803136 |  |  |
| LOC286059 | 4.923869586 | * | * |
| LNCTAM34A | 4.922636088 |  |  |
| LOC101928512 | 4.919307516 |  |  |
| WNT1 | 4.90591782 |  | * |
| INS-IGF2 | 4.900528644 | * | * |
| LRRN4 | 4.895582397 | * | * |
| LOC100129352 | 4.885058538 | * | * |
| CAV2 | 4.883210427 | * |  |
| RPL29P17 | 4.880458599 |  | * |
| LOC105378787 | 4.878352432 |  |  |
| LINC02051 | 4.875550211 | * | * |
| LOC101928675 | 4.86285255 | * | * |
| LOC105371781 | 4.85404272 |  |  |
| RN7SL181P | 4.842951612 | * |  |
| FAM201A | 4.838697171 |  | * |
| GBA3 | 4.83438131 | * | * |
| RPL5P30 | 4.832552664 |  | * |
| FOSB | 4.81221538 |  | * |
| SLC4A1 | 4.802848675 |  |  |
| LOC107984898 | 4.764800275 | * | * |
| LOC105374343 | 4.759815742 |  | * |
| LOC105379013 | 4.746773834 |  |  |
| IL1A | 4.743617458 | * | * |
| ELF5 | 4.741751123 | * | * |
| SNORD116-19 | 4.738269135 | * | * |
| HAPLN1 | 4.730003924 |  |  |
| LOC105370276 | 4.727017377 |  | * |
| CABP7 | 4.721740715 | * | * |
| LOC105371745 | 4.694096973 | * |  |
| CCDC65 | 4.679662252 |  | * |
| S100A7A | 4.673400851 | * | * |
| FOSL1 | 4.664968012 |  |  |
| RPL7AP45 | 4.663695639 | * | * |
| MUC4 | 4.663500137 |  |  |
| LOC112268260 | 4.662279996 | * | * |
| METTL21EP | 4.660291742 |  |  |
| LOC107984128 | 4.65775196 |  |  |
| LOC105376046 | 4.653090524 |  | * |
| TRIM64B | 4.647185311 |  | * |
| BMS1P2-AGAP9 | 4.633006248 | * | * |
| TRM-CAT6-1 | 4.632860334 | * | * |
| SEMA3G | 4.626659362 |  | * |
| HSPB2-C11orf52 | 4.621636554 | * |  |
| PRELID1P4 | 4.616588664 |  | * |
| GGT2 | 4.612082037 |  | * |
| TGFA | 4.600317037 |  |  |
| IL31RA | 4.595245762 |  |  |
| SOX18 | 4.590798159 |  | * |
| HCN2 | 4.568005106 |  | * |
| COMP | 4.558412885 | * | * |
| C4orf47 | 4.54962116 |  |  |
| MOG | 4.549439689 |  | * |
| LOC105378567 | 4.544877732 |  | * |
| LINC00322 | 4.543907187 | * | * |
| LOC102723490 | 4.539734231 |  |  |
| LOC112267987 | 4.521938698 |  |  |
| LOC107984862 | 4.519416239 | * | * |
| LOC107984751 | 4.504034357 |  |  |
| LOC105378577 | 4.490503041 | * | * |
| CTB-99A3.1 | 4.486910733 |  |  |
| TSPEAR | 4.475691231 | * |  |
| LOC105369959 | 4.474286527 |  |  |
| CALHM1 | 4.473398695 |  |  |
| LOC105373905 | 4.473390668 |  | * |
| LOC107984104 | 4.472884539 |  |  |
| BHMT2 | 4.471385508 | * |  |
| NPPC | 4.471230243 |  | * |
| GYG1P1 | 4.469310409 |  | * |
| LORICRIN | 4.461198117 | * |  |
| ALOX15B | 4.431187872 |  |  |
| HLA-F | 4.419043606 | * | * |
| MPP4 | 4.418144638 |  | * |
| PAPPA | 4.415312365 | * | * |
| MIR6748 | 4.412224503 | * |  |
| LINC01354 | 4.408472904 |  | * |
| SNORD140 | 4.405004634 |  | * |
| HPSE | 4.404187308 | * | * |
| LOC108783645 | 4.403611846 | * | * |
| LOC107984368 | 4.38933655 |  |  |
| SNORD108 | 4.388518316 | * | * |
| LOC101928530 | 4.384501113 | * | * |
| PCDHGA5 | 4.38442084 | * | * |
| ELF3 | 4.381773285 | * | * |
| EXD1 | 4.38019487 |  | * |
| BCL2L10 | 4.379570005 | * |  |
| NODAL | 4.364894288 | * | * |
| ZFP57 | 4.35137907 | * | * |
| LOC105374897 | 4.339347826 |  | * |
| LINC00311 | 4.338737807 |  | * |
| GABRA2 | 4.337562181 | * | * |
| RASD2 | 4.33180358 | * | * |
| TNFRSF8 | 4.330724697 | * | * |
| ANKRD18A | 4.328526226 |  |  |
| LINC00523 | 4.324617081 |  | * |
| SNORD116-9 | 4.322017084 | * |  |
| VTN | 4.317193589 | * | * |
| LINC01711 | 4.313856754 | * |  |
| LINC02072 | 4.311688919 | * |  |
| SCN10A | 4.306477316 | * | * |
| SBSN | 4.306404119 |  |  |
| CILP | 4.305281171 | * | * |
| F13A1 | 4.302265663 |  |  |
| TNFRSF10A | 4.297430142 |  |  |
| YWHABP1 | 4.29554753 |  | * |
| LINC01451 | 4.295038305 |  | * |
| MRGPRF | 4.288282084 |  |  |
| IFNL4 | 4.287702691 | * | * |
| CACYBPP2 | 4.266869986 |  |  |
| LOC105376544 | 4.263726453 |  | * |
| TRIM59-IFT80 | 4.260473 |  |  |
| RPL37A-DT | 4.258455391 | * |  |
| DENND5B-AS1 | 4.255558584 | * | * |
| PROCR | 4.254254081 |  |  |
| LOC112267954 | 4.245548229 |  | * |
| THSD1P1 | 4.24432167 |  | * |
| PABPC1L2B | 4.24309705 |  |  |
| TAS1R1 | 4.242159468 |  |  |
| MUC5B | 4.240587465 |  | * |
| FLJ32255 | 4.239696206 | * |  |
| LOC102724638 | 4.225051188 |  |  |
| SNORA63D | 4.22040867 |  |  |
| CIRBP-AS1 | 4.213677716 | * | * |
| MIR1204 | 4.208711903 | * | * |
| KRT19 | 4.195515042 | * | * |
| CDCP1 | 4.183737056 |  |  |
| RHOT1P2 | 4.179105106 | * | * |
| GAS6-DT | 4.153020272 |  | * |
| GPR132 | 4.14117379 |  |  |
| LOC100507516 | 4.139061422 |  |  |
| ENG | 4.130492637 | * | * |
| PHLDA2 | 4.128037578 |  |  |
| ANTXRL | 4.12634607 | * | * |
| LINC00513 | 4.126115235 |  | * |
| SLC12A8 | 4.120176071 |  | * |
| ARC | 4.118338128 |  |  |
| LOC107987159 | 4.116706016 | * |  |
| LOC105378910 | 4.116087204 | * | * |
| HNRNPKP2 | 4.112067347 | * |  |
| TRPM2 | 4.109177703 |  | * |
| MRPS30-DT | 4.101956789 |  | * |
| LOC102724734 | 4.101667697 |  |  |
| LOC112268253 | 4.099975088 | * | * |
| DOCK2 | 4.098519012 | * | * |
| LOC401805 | 4.093383138 | * |  |
| LOC105377382 | 4.093232728 |  |  |
| MIR1307 | 4.091795672 | * | * |
| ADIRF | 4.091568738 |  | * |
| FABP4 | 4.090665989 |  |  |
| LINC00598 | 4.08317256 |  | * |
| LOC107985716 | 4.082494029 | * | * |
| LOC101927394 | 4.081613968 |  |  |
| KCNA10 | 4.078230862 |  |  |
| SCTR-AS1 | 4.065095235 |  |  |
| CASC18 | 4.063853262 |  | * |
| LINC01204 | 4.056336038 |  |  |
| MIR3189 | 4.056266758 |  |  |
| MGP | 4.055540884 | * | * |
| PSMB8-AS1 | 4.048141011 | * |  |
| ESR1 | 4.0424396 | * | * |
| LINC01173 | 4.037831592 |  |  |
| LOC107984663 | 4.037180459 |  |  |
| LOC105379133 | 4.027515565 |  | * |
| LOC105373293 | 4.026809759 | * | * |
| TLR4 | 4.026283533 | * | * |
| CALML3 | 4.002008438 |  | * |
| FAM78B-AS1 | 4.00173512 |  | * |
| LINC00327 | 3.992169763 |  | * |
| EIF2S2P3 | 3.990277706 | * | * |
| C1QL3 | 3.985434434 | * |  |
| LOC105379244 | 3.982406558 |  |  |
| EEF1A1P3 | 3.976871611 | * | * |
| RPL23P8 | 3.975969713 | * | * |
| CNPY1 | 3.970375358 |  |  |
| LINC02703 | 3.964766839 | * | * |
| YBX1P2 | 3.964677383 | * | * |
| LOC107986664 | 3.960607881 |  |  |
| S100A3 | 3.957630228 | * | * |
| LOC105378628 | 3.956929778 | * |  |
| LOC102724830 | 3.949820068 |  |  |
| FAM131C | 3.949495293 | * |  |
| MIR3681HG | 3.947989986 |  | * |
| LOC107984118 | 3.940048538 |  | * |
| ANGPTL7 | 3.934361458 | * | * |
| LOC105369914 | 3.928418924 |  | * |
| KCNK15 | 3.92829889 | * | * |
| LOC105370998 | 3.92743547 |  |  |
| SLC6A11 | 3.924022735 |  | * |
| S100A10 | 3.92303994 |  |  |
| LOC105374378 | 3.921270205 |  |  |
| TRPV2 | 3.921213658 |  |  |
| FAM106A | 3.920429496 | * |  |
| HMGN2P12 | 3.908907351 |  | * |
| KCTD5P1 | 3.90231454 |  | * |
| PHOX2A | 3.901274143 | * | * |
| UBD | 3.896062019 | * |  |
| LOC105377378 | 3.893959231 |  |  |
| C6orf223 | 3.893133338 |  | * |
| TFR2 | 3.889543402 | * | * |
| KTI12 | 3.887864315 |  |  |
| NLRP5 | 3.887127644 |  | * |
| SUSD3 | 3.883596931 |  |  |
| TMPRSS6 | 3.881320541 | * | * |
| RNA5S3 | 3.878457178 | * | * |
| BRWD1-AS1 | 3.878124847 | * |  |
| LOC107987209 | 3.875858765 | * | * |
| ANKRD2 | 3.874557263 |  |  |
| LRRC70 | 3.873207423 | * | * |
| BDKRB2 | 3.863426799 |  | * |
| PPP1R14BP1 | 3.862696399 |  | * |
| ITIH5 | 3.862004265 |  |  |
| TBC1D29P | 3.860711424 |  | * |
| PRKAR1B-AS1 | 3.854646017 | * | * |
| CD81-AS1 | 3.854465364 |  |  |
| CCNP | 3.84642806 | * | * |
| TNFRSF18 | 3.837224961 | * |  |
| RPL26P6 | 3.836460274 | * |  |
| LAMC2 | 3.823735873 |  |  |
| UXT-AS1 | 3.816166455 |  | * |
| RPS19P3 | 3.815427448 |  | * |
| LOC107984928 | 3.815367519 | * |  |
| CFAP298-TCP10L | 3.812407434 |  | * |
| NUGGC | 3.811659193 |  |  |
| LOC107984877 | 3.810242142 | * |  |
| CDKN2B | 3.809280274 | * | * |
| DNAH5 | 3.804932161 | * |  |
| GOLGA6C | 3.800547945 | * | * |
| GPR17 | 3.800200344 |  |  |
| LUARIS | 3.797140857 |  | * |
| LINC02427 | 3.794547031 | * | * |
| ZNF670-ZNF695 | 3.792964892 | * | * |
| SNORA55 | 3.792793599 |  |  |
| LOC105371592 | 3.792792793 | * | * |
| LINC01629 | 3.789717711 |  | * |
| LOC107985715 | 3.785675193 | * | * |
| TDGF1P7 | 3.785198275 |  | * |
| LOC105369382 | 3.78340641 |  |  |
| ATP5PBP5 | 3.782550823 | * |  |
| SNORD77 | 3.778746532 | * |  |
| NR2E3 | 3.770950067 |  | * |
| LOC105371649 | 3.766351187 |  |  |
| LINC02716 | 3.764614875 |  |  |
| RPS9P2 | 3.762446282 |  | * |
| LINC02138 | 3.761786074 | * | * |
| PRAM1 | 3.759421121 |  | * |
| OR7E14P | 3.758435434 |  |  |
| LOC105373585 | 3.755262738 | * |  |
| LOC105378526 | 3.754658385 |  | * |
| LOC101929204 | 3.747786749 | * | * |
| LOC105378135 | 3.746216176 | * | * |
| KLK4 | 3.743074722 | * | * |
| LIF | 3.741067616 |  |  |
| MIR27B | 3.737864078 |  |  |
| SNORD18B | 3.73701765 | * | * |
| TRG-TCC2-3 | 3.736713634 |  | * |
| TRG-TCC2-5 | 3.736699016 |  | * |
| LOC101929710 | 3.736442236 | * |  |
| SLC2A9 | 3.734946274 |  | * |
| CNGA1 | 3.732034626 | * | * |
| SNORA78 | 3.727855301 | * | * |
| GPRC5A | 3.724682263 | * | * |
| LINC02725 | 3.7223588 | * |  |
| C11orf94 | 3.722316543 |  |  |
| HR | 3.720166485 | * | * |
| LOC105371731 | 3.719511802 | * | * |
| FYTTD1P1 | 3.717675713 | * | * |
| LOC101926984 | 3.715960117 |  |  |
| OXCT2 | 3.715517941 |  |  |
| LINC01770 | 3.715451368 |  |  |
| SPRY4-IT1 | 3.70820644 |  |  |
| SNORD89 | 3.701981436 | * | * |
| RNVU1-4 | 3.696037792 | * |  |
| LOC102724903 | 3.694657511 | * | * |
| MIR6751 | 3.687447515 |  |  |
| SP100 | 3.685130749 |  |  |
| LEFTY1 | 3.683778478 |  | * |
| PRIMA1 | 3.665962488 |  | * |
| SLC35G5 | 3.664057973 | * | * |
| NPR3 | 3.663045257 | * | * |
| TET2-AS1 | 3.662908631 |  |  |
| LINC01508 | 3.661297927 |  |  |
| ZNF726P1 | 3.659033689 | * | * |
| LEFTY2 | 3.65376901 | * | * |
| LOC102723911 | 3.653213374 |  |  |
| LOC105372838 | 3.647416947 |  | * |
| LOC102723313 | 3.6460566 | * | * |
| LANCL3 | 3.645981951 | * | * |
| LOC112267983 | 3.642544552 |  |  |
| LINC01999 | 3.641834409 | * |  |
| LOC107984272 | 3.641554098 |  | * |
| C19orf73 | 3.631190212 |  |  |
| LOC105377731 | 3.629481338 |  | * |
| PSMC1P3 | 3.627443315 | * |  |
| GLDN | 3.623802328 | * | * |
| TRIM22 | 3.622796705 |  |  |
| LOC102724146 | 3.622653022 | * | * |
| CORT | 3.621774447 | * |  |
| MYCLP1 | 3.610622856 |  | * |
| NNMT | 3.595099175 |  | * |
| ANXA1 | 3.594190156 |  |  |
| LOC101927383 | 3.592006441 |  |  |
| LOC105371730 | 3.577590443 |  |  |
| MIR6764 | 3.573959882 |  | * |
| GDNF-AS1 | 3.573342051 | * |  |
| LOC105369533 | 3.566931531 |  |  |
| APOL6 | 3.56537679 |  |  |
| LOC105376484 | 3.563606456 |  |  |
| PIR-FIGF | 3.56318597 | * | * |
| C5orf46 | 3.561344373 |  |  |
| CAVIN1 | 3.557442222 |  |  |
| FAM83E | 3.553881254 | * | * |
| MYO1G | 3.546708599 |  |  |
| BIRC3 | 3.54603841 |  |  |
| TMEM236 | 3.545536869 |  | * |
| MIR937 | 3.545527688 |  | * |
| LOC105372814 | 3.54404485 |  |  |
| NEUROG2 | 3.543211884 | * | * |
| ZMIZ1-AS1 | 3.540969352 | * | * |
| LOC100287049 | 3.540475555 | * |  |
| LOC110091768 | 3.538633578 |  |  |
| LGSN | 3.53696785 | * |  |
| LOC105370525 | 3.532415851 | * | * |
| PTPN7 | 3.530386849 | * | * |
| ANKRD49P1 | 3.528181097 |  | * |
| CNGA3 | 3.527602571 |  | * |
| LOC107987042 | 3.519710468 |  | * |
| CYP4F35P | 3.515804886 |  |  |
| LOC101927834 | 3.509211764 | * | * |
| MIR6843 | 3.508865656 |  |  |
| PKDREJ | 3.507902299 |  |  |
| NXT1-AS1 | 3.502631952 | * | * |
| KCNQ1DN | 3.494535103 | * | * |
| SMIM35 | 3.493608731 |  | * |
| CLDN6 | 3.491988633 |  |  |
| FLT3LG | 3.491850841 | * |  |
| ITGA11 | 3.488787424 | * | * |
| LOC107984852 | 3.483793108 |  |  |
| CLEC4A | 3.482942641 | * |  |
| LOC101928502 | 3.481747207 |  | * |
| LOC100287387 | 3.481367845 | * | * |
| LOC101927824 | 3.478723453 |  |  |
| LOC105377632 | 3.473630854 |  | * |
| INPP5D | 3.473036494 |  | * |
| OPTC | 3.466543398 |  | * |
| SNORD16 | 3.464696869 |  | * |
| TMPRSS9 | 3.463751252 | * | * |
| ZNF396 | 3.46364672 | * | * |
| CT66 | 3.462125289 |  |  |
| SYNE1-AS1 | 3.461599769 |  | * |
| LOC107984837 | 3.458429033 |  | * |
| BTNL2 | 3.456791611 | * | * |
| LOC107984591 | 3.455451574 |  | * |
| LOC105369583 | 3.454719206 |  | * |
| SUB1P1 | 3.452664612 |  |  |
| LOC107984833 | 3.450014693 | * | * |
| HMGN2P46 | 3.449758443 | * |  |
| HKDC1 | 3.44925148 |  |  |
| ALDH8A1 | 3.448152577 | * |  |
| PARVG | 3.443627639 |  |  |
| NIPAL1 | 3.442390588 | * |  |
| LOC105369534 | 3.440254635 |  |  |
| LOC91450 | 3.435890045 |  |  |
| ADAMTS15 | 3.433232349 |  |  |
| LUCAT1 | 3.432906894 | * | * |
| TNFRSF1B | 3.429795368 | * | * |
| FOXA3 | 3.426686515 | * |  |
| LOC105374291 | 3.425610688 |  | * |
| MICALCL | 3.421990133 |  | * |
| PXT1 | 3.420497388 | * | * |
| LOC112268149 | 3.418004392 | * | * |
| SNORD33 | 3.417919125 | * |  |
| LOC441711 | 3.417395945 |  |  |
| SEZ6 | 3.413799617 | * | * |
| MFAP5 | 3.412335981 |  |  |
| HCST | 3.406308344 |  |  |
| TNFRSF11B | 3.406184946 | * |  |
| ST13P6 | 3.405163253 |  | * |
| LOC105374306 | 3.398721885 |  |  |
| ANKRD1 | 3.395072504 |  |  |
| RNA5S4 | 3.39479428 | * | * |
| LOC107984224 | 3.392640842 |  |  |
| RNU6-11P | 3.384784563 | * | * |
| LOC107986548 | 3.382153533 |  | * |
| LOC107984655 | 3.381967801 |  |  |
| LINC02277 | 3.381812526 | * |  |
| LOC107987237 | 3.381760667 | * | * |
| MMP19 | 3.381474549 | * |  |
| LOC105374775 | 3.36650989 | * |  |
| DCXR-DT | 3.356660398 |  |  |
| FOXD4L5 | 3.353473079 |  | * |
| LOC105371159 | 3.343906802 |  | * |
| CSMD1 | 3.342364532 |  |  |
| GJB2 | 3.340581454 | * | * |
| ASPHD1 | 3.339931984 |  |  |
| ABCF1-DT | 3.33619292 | * |  |
| LOC107984435 | 3.329457268 | * | * |
| TM4SF1 | 3.325759191 | * | * |
| SCN5A | 3.323778786 |  |  |
| LINC01250 | 3.323225863 |  |  |
| RANBP3L | 3.322218927 | * | * |
| LOC105372708 | 3.321879727 |  |  |
| ARRDC4 | 3.321664375 |  |  |
| LOC100134317 | 3.314496937 |  |  |
| RPL15P22 | 3.3113056 | * |  |
| GLIPR1 | 3.310331053 | * | * |
| SEMA3F-AS1 | 3.308869158 | * | * |
| TMEM217 | 3.306694032 | * | * |
| FAM95C | 3.3063876 |  | * |
| GP5 | 3.304655093 |  |  |
| LOC107985729 | 3.304101033 |  | * |
| LOC105371393 | 3.302234747 |  | * |
| SGMS2 | 3.301096769 |  |  |
| LOC284240 | 3.2999727 |  |  |
| H2BU2P | 3.299793148 |  | * |
| LINC01512 | 3.298296942 |  |  |
| CIITA | 3.296674749 |  |  |
| HBA2 | 3.293235449 |  |  |
| HAVCR1 | 3.292936175 |  |  |
| MIR10399 | 3.292391845 |  | * |
| SNORD25 | 3.289711781 | * | * |
| LOC107984906 | 3.288836175 | * | * |
| MIR210 | 3.288785327 |  |  |
| GOLT1A | 3.285227218 |  |  |
| LOC105370774 | 3.284259521 | * |  |
| WNT10B | 3.283521358 |  | * |
| LINC02551 | 3.280243031 |  | * |
| SPATA18 | 3.279566969 |  |  |
| ZACN | 3.277734502 | * | * |
| SSTR5 | 3.274120538 | * | * |
| NLRC5 | 3.269982317 |  |  |
| SSTR4 | 3.268084959 |  |  |
| CELSR1 | 3.26747043 |  | * |
| CD274 | 3.267234856 | * |  |
| RTN3P1 | 3.259931533 | * |  |
| NYAP2 | 3.258249907 | * |  |
| LOC105370027 | 3.258021516 |  |  |
| TENT5A | 3.252876681 | * | * |
| C15orf56 | 3.250813681 | * | * |
| TPSAB1 | 3.246388856 |  | * |
| HABP2 | 3.246002649 |  |  |
| H2BU1 | 3.240552167 |  | * |
| GDNF | 3.238113066 |  |  |
| LAYN | 3.237340519 |  |  |
| LOC100505909 | 3.236828364 |  | * |
| LINC01587 | 3.231976633 | * | * |
| SLC6A5 | 3.231334739 | * | * |
| SPTA1 | 3.227621029 |  | * |
| ARHGDIB | 3.226429844 | * | * |
| ENPP7 | 3.222732082 |  | * |
| LOC107986065 | 3.216133294 |  | * |
| SBK3 | 3.209235973 |  |  |
| LOC105373027 | 3.20910699 |  | * |
| LOC724084 | 3.207804839 | * |  |
| LINC01703 | 3.205954423 |  |  |
| LOC105373176 | 3.205382411 |  |  |
| METTL7B | 3.200837315 |  |  |
| MIR6890 | 3.199688137 |  | * |
| LOC105373414 | 3.197926547 | * |  |
| LOC107986477 | 3.197883174 | * | * |
| KANSL1-AS1 | 3.191187657 |  |  |
| SNORD42B | 3.184246719 | * | * |
| RASGRF1 | 3.181646492 |  |  |
| ROPN1L | 3.180629299 | * |  |
| LOC107986641 | 3.17647669 |  | * |
| DLX3 | 3.175140684 |  | * |
| RPS15AP14 | 3.174397555 |  | * |
| NEUROG2-AS1 | 3.172601288 | * | * |
| NDUFAF4P3 | 3.171556284 | * | * |
| NPY4R2 | 3.169635941 |  |  |
| MYRFL | 3.158382069 |  | * |
| LOC101929216 | 3.156086853 |  | * |
| PLAU | 3.153992594 |  |  |
| LASTR | 3.153455024 |  |  |
| VWF | 3.153019424 |  |  |
| CSGALNACT1 | 3.151951031 |  | * |
| ALPP | 3.151036955 | * | * |
| THORLNC | 3.1474116 |  |  |
| EDA2R | 3.146167335 |  |  |
| LOC105369952 | 3.144695956 | * | * |
| LOC107985452 | 3.144182391 | * |  |
| SEMA3F | 3.14379627 |  | * |
| TMIE | 3.140021951 | * | * |
| ANO2 | 3.137727613 |  | * |
| RAB20 | 3.135010723 |  |  |
| FRK | 3.12900348 | * | * |
| TMEM163 | 3.12509428 |  |  |
| LOC105374260 | 3.124996038 |  |  |
| LOC107984868 | 3.115439117 | * | * |
| HRAT17 | 3.110627939 |  |  |
| USP12-AS2 | 3.107475121 | * |  |
| LOC107983985 | 3.104390603 | * |  |
| PI15 | 3.098897549 | * | * |
| SNORD116-29 | 3.092720887 |  |  |
| KCNK6 | 3.09242096 |  |  |
| KLF3P1 | 3.089775561 |  |  |
| TEKT5 | 3.089498946 |  | * |
| FAM230D | 3.088821515 |  | * |
| KCNB1 | 3.08729076 |  |  |
| LINC01562 | 3.08551974 |  |  |
| MSANTD1 | 3.085185737 | * | * |
| BIRC7 | 3.084288505 |  |  |
| CD8A | 3.084012656 | * | * |
| DNAH2 | 3.08228048 | * | * |
| LOC100126784 | 3.081367837 |  |  |
| MIR34AHG | 3.081310517 |  |  |
| GPAA1P1 | 3.08015616 | * | * |
| LINC02678 | 3.074596411 |  | * |
| HAPLN3 | 3.071946314 |  | * |
| LOC101927863 | 3.068661364 | * | * |
| CCDC39 | 3.061908244 |  | * |
| ATP2B3 | 3.059096547 |  | * |
| NSA2P2 | 3.059072361 |  |  |
| TRG-CCC2-2 | 3.055307129 |  |  |
| OLA1P1 | 3.053054662 | * | * |
| LOC112268415 | 3.049219801 | * | * |
| ALMS1-IT1 | 3.049215978 |  | * |
| RPL37AP5 | 3.045307313 | * | * |
| BNC1 | 3.04383148 |  |  |
| NUTM2G | 3.041980996 |  |  |
| ADGRA1-AS1 | 3.038101483 |  | * |
| WBP1LP2 | 3.037485558 |  |  |
| FAM106B | 3.035620364 | * | * |
| SH3RF3-AS1 | 3.032507874 |  | * |
| LOC101929894 | 3.031003527 |  |  |
| MYCT1 | 3.030641837 |  | * |
| GDF6 | 3.029244314 | * | * |
| C1orf115 | 3.027890382 |  |  |
| LOC100289333 | 3.027859478 |  |  |
| PABPN1L | 3.027324492 |  | * |
| RPL35P2 | 3.02586105 | * | * |
| ARSJ | 3.024919311 | * | * |
| ABCB11 | 3.023828363 | * | * |
| ANXA3 | 3.022400107 |  |  |
| LOC107986114 | 3.021746805 |  |  |
| LOC105369820 | 3.021631036 | * |  |
| LOC107986704 | 3.016414291 |  |  |
| TGFB1 | 3.016235105 | * | * |
| GCK | 3.01519839 |  |  |
| LOC105374952 | 3.012787698 |  |  |
| GXYLT1P4 | 3.010443864 |  | * |
| IL7R | 3.005983545 |  | * |
| LOXL4 | 3.005349229 |  | * |
| LOC105378629 | 3.004788742 | * | * |
| SBK2 | 3.004549817 |  |  |
| LOC105375905 | 3.004258518 |  |  |
| LINC01836 | 3.004187541 |  |  |
| MIR4730 | 3.001350443 | * |  |
| TRG-TCC2-4 | 2.998462523 |  |  |
| LOC107986110 | 2.996330275 | * | * |
| H2AC4 | 2.99631422 |  | * |
| HSP90AA3P | 2.995409457 | * |  |
| GJA9-MYCBP | 2.994064246 |  |  |
| RPS17P16 | 2.992723687 | * |  |
| KDM2B-DT | 2.991492689 |  |  |
| POM121L9P | 2.991277051 | * | * |
| PKNOX2-AS1 | 2.990162261 | * | * |
| TCIM | 2.988788306 | * |  |
| KRT80 | 2.98621886 |  |  |
| ANO4 | 2.984656374 |  |  |
| TIAF1 | 2.974587372 | * |  |
| LOC101928505 | 2.974301897 | * | * |
| ADAP1 | 2.972108658 | * | * |
| GSDMC | 2.966136158 | * | * |
| S100A6 | 2.962595039 |  |  |
| SLC6A17 | 2.962232517 | * | * |
| TGM5 | 2.962175357 |  | * |
| P2RY6 | 2.960840128 | * | * |
| FAT2 | 2.960547306 |  | * |
| JAKMIP1 | 2.958300028 |  |  |
| LOC101927506 | 2.957732043 | * |  |
| RGS4 | 2.957493909 | * | * |
| CD34 | 2.956060956 | * | * |
| EEF1B2P2 | 2.955388165 |  |  |
| OR7E37P | 2.953438121 | * | * |
| ELF4 | 2.951209278 |  |  |
| CD55 | 2.95066445 | * | * |
| VKORC1P2 | 2.950651716 |  | * |
| C2orf66 | 2.9502181 |  |  |
| HAUS6P1 | 2.949947695 |  | * |
| MYBPHL | 2.946891428 |  | * |
| VSIR | 2.945347008 |  |  |
| LOC105378054 | 2.944518187 |  |  |
| SLC17A8 | 2.943769529 |  |  |
| LOC105371532 | 2.943657625 |  |  |
| ANKRD33B | 2.940567492 |  |  |
| HAL | 2.940503331 |  |  |
| CCDC27 | 2.940321912 |  |  |
| LOC105373283 | 2.938476819 | * | * |
| SNTG2 | 2.937555436 |  |  |
| LINC00702 | 2.937233987 |  |  |
| LOC728739 | 2.937183884 |  |  |
| RPL7AP62 | 2.936834136 | * |  |
| FCAMR | 2.935696667 |  |  |
| IGFBP3 | 2.932823165 | * | * |
| MMP9 | 2.932263515 |  |  |
| LOC105375942 | 2.929718011 | * | * |
| VDR | 2.928055245 |  | * |
| FGD5 | 2.923832658 |  | * |
| LOC107984689 | 2.92132274 |  |  |
| CTB-30L5.1 | 2.919180351 |  |  |
| RN7SL521P | 2.918864396 | * | * |
| PNPLA5 | 2.918064116 | * | * |
| EGR3 | 2.916859202 |  |  |
| LOC105376381 | 2.916306538 |  |  |
| BMP8B | 2.915677837 |  |  |
| IGFALS | 2.915367803 |  |  |
| CCDC3 | 2.911810839 | * | * |
| LOC105375503 | 2.911736046 |  | * |
| RNA5S1 | 2.911098804 | * | * |
| POM121L1P | 2.910979526 |  |  |
| SNORD3B-1 | 2.907707502 |  |  |
| MIR6832 | 2.907689533 |  |  |
| LOC105378157 | 2.905109755 | * |  |
| FOXD1 | 2.904643301 | * |  |
| LOC102724545 | 2.899688016 |  | * |
| LOC105370876 | 2.897024142 |  | * |
| HRH3 | 2.895463993 | * | * |
| LINC00987 | 2.894933865 | * |  |
| COL15A1 | 2.894857066 |  | * |
| PAK6 | 2.89421892 |  |  |
| SLC25A45 | 2.89342192 |  |  |
| SNORA103 | 2.893249425 |  |  |
| LOC100134391 | 2.892347146 |  | * |
| AQP7 | 2.891531509 |  | * |
| LOC105373415 | 2.891225763 |  | * |
| LINC02561 | 2.890733734 |  |  |
| MPPED1 | 2.88614549 | * | * |
| CMKLR1 | 2.885659804 |  | * |
| CCN2 | 2.884483748 | * | * |
| LOX | 2.883072993 |  |  |
| LINC02714 | 2.882189348 |  | * |
| BHLHE40 | 2.880501464 |  |  |
| MIR6777 | 2.880422832 |  |  |
| OSBPL3 | 2.878083596 |  |  |
| MARCHF4 | 2.87609009 |  | * |
| LMX1B | 2.875294053 |  | * |
| SLC37A2 | 2.874444777 | * | * |
| ENPP2 | 2.872107573 |  |  |
| CYP19A1 | 2.872069161 | * | * |
| KCNJ5 | 2.871310812 |  |  |
| MIR193BHG | 2.870340487 |  | * |
| MMP17 | 2.862893735 |  | * |
| ADAMTS8 | 2.86166872 |  |  |
| PAXBP1-AS1 | 2.861359027 | * |  |
| MMP28 | 2.861157082 |  | * |
| SNORA17B | 2.860887728 | * | * |
| LRRK1 | 2.860223179 |  | * |
| EPO | 2.859922202 | * | * |
| ARAP2 | 2.859427801 | * | * |
| TDRD6 | 2.859078976 | * | * |
| FRG1FP | 2.854262579 |  | * |
| FAS | 2.852873757 |  |  |
| KCNAB1 | 2.852637819 |  |  |
| IGFN1 | 2.852117325 | * | * |
| ERP27 | 2.850919329 | * | * |
| C1GALT1C1L | 2.850491234 |  | * |
| CXCL6 | 2.848124216 | * |  |
| LOC107985054 | 2.845551781 |  | * |
| GGTLC2 | 2.843938807 |  |  |
| LOC105369180 | 2.843847688 |  | * |
| KRT18 | 2.842335586 |  |  |
| ARHGEF16 | 2.841700214 | * | * |
| CD9 | 2.841027556 |  |  |
| LOC347097 | 2.839831662 | * | * |
| HBEGF | 2.839360645 |  |  |
| IL32 | 2.830288041 | * | * |
| CLU | 2.827877712 |  |  |
| LOC107986639 | 2.826699744 |  |  |
| LOC101927793 | 2.826242139 |  | * |
| BMP8A | 2.8261363 |  | * |
| DUSP9 | 2.825627221 |  | * |
| ADGRF4 | 2.823362695 | * | * |
| CDA | 2.819443385 | * | * |
| SLC2A5 | 2.81830845 |  |  |
| ESRRAP2 | 2.817985693 | * |  |
| MIR4435-2HG | 2.814160803 |  |  |
| ADAMTS2 | 2.812519433 |  |  |
| SLC17A9 | 2.803322849 |  |  |
| LAMA3 | 2.802326237 |  | * |
| SH2D3A | 2.800707772 | * | * |
| MIR570 | 2.799408086 | * | * |
| PIGY-DT | 2.797518951 | * |  |
| LHX4 | 2.794157547 | * | * |
| LOC105378963 | 2.7920929 |  | * |
| GDF7 | 2.791453485 | * | * |
| ARHGDIG | 2.790878848 | * |  |
| LOC105369293 | 2.790775241 |  |  |
| EMSLR | 2.790378335 |  |  |
| ROS1 | 2.790371433 | * |  |
| AIDAP2 | 2.788250211 |  |  |
| LOC102724805 | 2.787607886 |  | * |
| LINC01783 | 2.786655331 | * | * |
| GCNT4 | 2.786576105 |  |  |
| LOC107986862 | 2.785414374 |  | * |
| DAPP1 | 2.783542163 | * |  |
| WFDC1 | 2.782970886 |  | * |
| GABRB1 | 2.781512111 | * | * |
| LOC101927040 | 2.779688162 |  | * |
| SNORA20 | 2.779201977 | * |  |
| TRPC2 | 2.77888155 |  |  |
| PDZK1 | 2.777097247 |  | * |
| MIR6516 | 2.774607759 |  | * |
| LOC105376952 | 2.774128744 | * |  |
| TACSTD2 | 2.771705465 |  | * |
| MRPL23-AS1 | 2.771189562 |  |  |
| VNN2 | 2.771019965 | * | * |
| TBX20 | 2.769613397 |  |  |
| LOC100652777 | 2.767891807 |  | * |
| GALNT15 | 2.767396938 |  | * |
| LOC105371175 | 2.767323486 | * | * |
| AMDHD1 | 2.766120189 |  |  |
| DLK2 | 2.762894182 |  | * |
| FOXD4L4 | 2.762497375 |  |  |
| LINC01186 | 2.760295205 |  |  |
| LOC105379251 | 2.757691236 |  |  |
| NANOS1 | 2.756613215 |  | * |
| AQP8 | 2.755401326 |  |  |
| SDC4 | 2.755236956 |  |  |
| LCN2 | 2.754663509 | * | * |
| IL15 | 2.754212811 |  |  |
| LOC105375124 | 2.753951934 |  |  |
| CCND1 | 2.753468901 |  |  |
| TMC8 | 2.752201622 |  |  |
| PPP2R2B | 2.752163159 |  |  |
| LOC105372692 | 2.749095191 | * | * |
| RNU4ATAC | 2.746148156 |  | * |
| DLGAP3 | 2.745878871 |  |  |
| LOC105373785 | 2.743127874 | * | * |
| IGFBP7 | 2.74098588 | * | * |
| GNAT1 | 2.738596713 |  | * |
| IGFBP6 | 2.738466681 |  |  |
| MAGEB3 | 2.737578996 |  | * |
| NBPF5P | 2.736215842 |  |  |
| PLPP2 | 2.736112263 |  |  |
| SBF2-AS1 | 2.734735592 | * | * |
| EEF1A1P8 | 2.734687442 |  |  |
| ABCB5 | 2.731893266 |  |  |
| LOC100128653 | 2.730415203 |  |  |
| SNORD116-14 | 2.7298446 | * |  |
| CTBP2P4 | 2.728908871 |  | * |
| LOC112268126 | 2.72543901 |  |  |
| BARX1-DT | 2.723741677 |  |  |
| LINC02698 | 2.721759673 | * | * |
| LRRC3-DT | 2.719358654 |  | * |
| LOC107984245 | 2.717867879 |  |  |
| PHLDA3 | 2.715330646 |  |  |
| NOL4 | 2.715290926 |  |  |
| LOC101928674 | 2.713893263 |  | * |
| LOC100132570 | 2.709644316 |  |  |
| TPRG1 | 2.708049895 | * | * |
| ADAM11 | 2.707635368 | * | * |
| RRAD | 2.706254371 | * | * |
| CALB1 | 2.705789089 | * |  |
| NPIPB10P | 2.700868572 |  |  |
| CCDC187 | 2.700304704 | * | * |
| TFCP2L1 | 2.696723793 |  |  |
| LOC107986613 | 2.694387062 | * |  |
| LOC107987233 | 2.694307779 | * | * |
| TFAP2E | 2.692410266 |  | * |
| CH25H | 2.691250138 |  |  |
| LOC107987334 | 2.690604913 |  | * |
| TESC | 2.690178425 | * | * |
| TMEM184A | 2.689948042 | * | * |
| LOC107984830 | 2.688258052 | * | * |
| HTRA1 | 2.685576266 |  | * |
| LOC105373977 | 2.68537243 |  |  |
| LOC102724892 | 2.685001469 |  |  |
| BST1 | 2.681123995 |  | * |
| LOC105371168 | 2.680327727 | * | * |
| ZNF687-AS1 | 2.678053242 |  |  |
| GBP2 | 2.677376336 |  |  |
| LINC02777 | 2.676959362 |  | * |
| TC2N | 2.675488735 | * | * |
| TAFA3 | 2.674540124 |  | * |
| SNORA33 | 2.673521382 | * | * |
| USH2A | 2.672424951 |  |  |
| LOC107986673 | 2.672393639 | * | * |
| LOC105371216 | 2.671573359 | * | * |
| KCNK7 | 2.671332169 |  |  |
| PLCH2 | 2.671009757 |  |  |
| SRPX2 | 2.671001221 |  | * |
| C8orf89 | 2.669925966 |  | * |
| ERMN | 2.669234061 |  |  |
| LOC105369246 | 2.669189993 | * | * |
| ALX3 | 2.668975218 | * | * |
| LOC105369323 | 2.668293744 |  |  |
| LOC100130872 | 2.667040083 |  | * |
| MIR10393 | 2.666214104 | * | * |
| WNT2B | 2.66586583 |  |  |
| LOC105373282 | 2.665711633 | * | * |
| LOC107985362 | 2.663968662 |  |  |
| H3C1 | 2.663382305 |  |  |
| MAPK6P3 | 2.6632451 |  | * |
| LOC100134868 | 2.661740937 |  |  |
| LOC105371795 | 2.659263822 | * | * |
| PPM1N | 2.654622735 |  | * |
| NOP53-AS1 | 2.65133112 |  | * |
| FMC1-LUC7L2 | 2.650521289 | * | * |
| NFE2L3P2 | 2.648937671 | * | * |
| SHANK2 | 2.647668164 |  |  |
| LOC101928576 | 2.647440936 | * |  |
| RNF152P1 | 2.647430608 |  |  |
| LOC105375114 | 2.644173411 |  |  |
| CA3 | 2.644102378 |  |  |
| LINC02885 | 2.641085618 |  |  |
| ANKDD1B | 2.640859041 | * | * |
| CARD6 | 2.635713246 |  |  |
| LOC105371629 | 2.634765044 |  |  |
| MIR6746 | 2.633597486 |  | * |
| ADAMTSL1 | 2.631002888 |  |  |
| LOC101929307 | 2.630989957 |  | * |
| LOC105373944 | 2.629851512 |  |  |
| TNK2-AS1 | 2.629024462 | * | * |
| GCC2-AS1 | 2.628558828 | * | * |
| LOC107984391 | 2.624295162 |  |  |
| SNORD51 | 2.62334752 | * |  |
| LOC101927550 | 2.622193335 |  |  |
| RPL21P133 | 2.620563905 |  | * |
| MAFK | 2.618351939 |  | * |
| LOC105369209 | 2.617440758 |  | * |
| MIR611 | 2.617218021 |  | * |
| FADS3 | 2.616034141 |  |  |
| PAX9 | 2.61602511 | * |  |
| LOC105373873 | 2.614547285 |  |  |
| TMEM154 | 2.612485264 |  |  |
| LOC105374128 | 2.6117627 |  | * |
| BHLHA15 | 2.611683374 | * | * |
| SCG2 | 2.606885133 |  | * |
| POU6F2-AS2 | 2.602845988 |  |  |
| SLC5A1 | 2.6026927 |  |  |
| LOC101927467 | 2.599864008 | * | * |
| ATG9B | 2.599385104 |  | * |
| MIR6513 | 2.598865999 | * | * |
| LOC105379242 | 2.596962077 |  | * |
| CD52 | 2.595863106 | * | * |
| SLC8A2 | 2.594908408 |  |  |
| ZP1 | 2.594332669 | * | * |
| HTR3E | 2.593907758 | * | * |
| LINC00304 | 2.593037527 |  | * |
| SMU1P1 | 2.592012107 | * | * |
| GRM5 | 2.590020245 |  | * |
| RN7SL689P | 2.587688225 |  | * |
| FBXL16 | 2.585131894 |  |  |
| FCRLA | 2.58391688 | * | * |
| LOC105377876 | 2.583833493 |  |  |
| CYYR1-AS1 | 2.581048639 |  | * |
| RASAL1 | 2.578943837 | * | * |
| LINC00466 | 2.578726478 | * |  |
| CHIT1 | 2.576934923 | * | * |
| KSR2 | 2.576834627 |  | * |
| AIRE | 2.576797682 |  |  |
| SLC25A34-AS1 | 2.576534817 |  |  |
| CD163 | 2.576391509 |  |  |
| FRMD5 | 2.575599829 |  |  |
| LINC01611 | 2.574107169 |  |  |
| SLC31A1P1 | 2.573586872 |  |  |
| NGF | 2.56931615 |  |  |
| CAV1 | 2.567503686 |  |  |
| COL12A1 | 2.567006262 |  | * |
| EDIL3-DT | 2.566842404 |  |  |
| MMP24 | 2.564474318 |  | * |
| ANKRD18B | 2.564421202 |  |  |
| TIAM2 | 2.564379428 |  | * |
| MIR135A2 | 2.56308671 |  |  |
| LOC105371206 | 2.562943937 | * | * |
| S100Z | 2.562034056 |  |  |
| MALINC1 | 2.559323186 |  | * |
| HSPB6 | 2.558343362 | * | * |
| ECM1 | 2.557802687 |  |  |
| LOC100505555 | 2.557595589 | * | * |
| LOC105371196 | 2.552898115 |  |  |
| LINC01606 | 2.550054885 |  | * |
| TXNIP | 2.549424197 |  |  |
| AMZ1 | 2.548734912 |  |  |
| LOC105376188 | 2.544116712 |  |  |
| B3GNT4 | 2.543745739 | * | * |
| CCDC170 | 2.540773562 |  |  |
| RRM2B | 2.540325792 |  |  |
| LOC107984529 | 2.537315658 | * |  |
| ESRP1 | 2.536890703 |  |  |
| LINC02799 | 2.536846736 |  |  |
| LINC00051 | 2.536582972 |  | * |
| LOC105379163 | 2.535808949 | * |  |
| LOC102724646 | 2.535025883 |  |  |
| KRTAP10-7 | 2.534771383 |  |  |
| RPSAP45 | 2.53248336 | * | * |
| HCRT | 2.532059501 |  |  |
| LONRF3 | 2.531585788 | * | * |
| GREM1 | 2.531507016 | * | * |
| LOC105375782 | 2.531264659 | * | * |
| TMEM132B | 2.531215593 |  |  |
| SLC39A4 | 2.530593199 |  |  |
| DYRK3-AS1 | 2.530568863 | * | * |
| LOC105372888 | 2.528517745 |  |  |
| GTSE1-DT | 2.521676068 |  |  |
| LMCD1-AS1 | 2.521478918 |  |  |
| C12orf75 | 2.521009153 |  |  |
| LOC101929418 | 2.519581783 |  |  |
| LOC105374615 | 2.51831172 | * |  |
| ADGRB1 | 2.51710854 | * | * |
| LINC02463 | 2.516693008 |  | * |
| RNF217-AS1 | 2.516113546 |  |  |
| LINC00593 | 2.51599883 | * | * |
| TRPV1 | 2.513523237 |  |  |
| SLITRK4 | 2.513187348 | * |  |
| ZMYND12 | 2.513148418 |  |  |
| H2AW | 2.512358884 |  | * |
| LOC102723401 | 2.511330701 |  | * |
| ME1 | 2.510733824 | * | * |
| ZMAT3 | 2.510013813 |  |  |
| LOC107985044 | 2.508909594 |  |  |
| SEMA3E | 2.508602994 |  |  |
| XBP1P1 | 2.508471532 | * | * |
| GYS2 | 2.507868167 |  |  |
| FSIP2-AS2 | 2.507808555 |  | * |
| TINAGL1 | 2.507582603 | * | * |
| MAMDC2 | 2.507226176 |  |  |
| PRKN | 2.506985591 | * | * |
| LOC105378570 | 2.506296817 | * | * |
| GGTLC5P | 2.505790905 |  |  |
| IL6R-AS1 | 2.504813351 | * | * |
| AQP1 | 2.503082051 |  |  |
| GIPR | 2.502487604 |  |  |
| MIR6741 | 2.502409844 | * |  |
| FAM198B-AS1 | 2.502172086 |  |  |
| LOC101927245 | 2.501736801 |  |  |
| CAVIN3 | 2.499923926 |  |  |
| GGT5 | 2.499459295 |  |  |
| MIR378I | 2.499340891 |  | * |
| LOC105376020 | 2.498820552 | * | * |
| LOC101927888 | 2.496239859 | * | * |
| LOC105369957 | 2.495631739 |  |  |
| SLC35F4 | 2.49500087 |  |  |
| LOC728752 | 2.494409766 |  |  |
| RUNDC3A-AS1 | 2.492810355 |  | * |
| SVOP | 2.492501532 |  |  |
| LOC101929314 | 2.491913237 | * | * |
| HIF1A-AS3 | 2.489918232 |  |  |
| LOC101926969 | 2.488165502 | * | * |
| CCDC151 | 2.487883743 | * |  |
| NIPAL4 | 2.487700952 |  | * |
| LINC01137 | 2.486328615 |  |  |
| MAP3K14-AS1 | 2.484199721 |  |  |
| P2RX5-TAX1BP3 | 2.483525711 | * | * |
| NR0B1 | 2.480975007 | * |  |
| LOC112268150 | 2.480797324 | * |  |
| LNX1 | 2.478620051 |  |  |
| IRAK2 | 2.478540626 |  | * |
| RET | 2.475936351 |  | * |
| SLC22A20P | 2.473789859 |  |  |
| LOC102725065 | 2.470826244 | * | * |
| LOC101929343 | 2.470603049 | * | * |
| FBLN5 | 2.469321084 |  |  |
| IGFBP7-AS1 | 2.467493701 | * |  |
| MIR214 | 2.46687054 | * | * |
| INHBA | 2.466680989 | * | * |
| LINC00857 | 2.465873105 |  |  |
| PLEKHG7 | 2.463341641 |  |  |
| KISS1 | 2.462427906 |  | * |
| NUAK1 | 2.462413427 |  |  |
| LOC105371055 | 2.461787658 |  |  |
| KRT18P17 | 2.461595543 |  |  |
| FAM90A12P | 2.459963695 |  |  |
| CNN1 | 2.457664041 | * | * |
| LOC105373184 | 2.457282604 | * | * |
| NFE2L3 | 2.457171889 |  |  |
| LOC401557 | 2.456377171 | * | * |
| FEM1AP2 | 2.456200949 |  |  |
| TMEM233 | 2.456094522 |  |  |
| UBE2D3P2 | 2.455867082 |  |  |
| LOC105373174 | 2.453922868 | * | * |
| MYL7 | 2.453839042 | * | * |
| BMS1P13 | 2.450984355 |  |  |
| LOC105375010 | 2.450681173 | * | * |
| CYTOR | 2.450452887 |  |  |
| LOC105373608 | 2.449769504 | * | * |
| LOC105369329 | 2.449145093 | * | * |
| GMFG | 2.448987065 | * |  |
| LOC105369761 | 2.447140623 | * | * |
| MIR12128 | 2.446401254 |  | * |
| LOC107987024 | 2.445582522 |  |  |
| TNFAIP8L2-SCNM1 | 2.444853384 |  | * |
| KIAA0040 | 2.444400684 | * | * |
| LOC105373311 | 2.443429129 |  |  |
| LOC653160 | 2.442899218 |  |  |
| HECW2-AS1 | 2.440913418 |  |  |
| PADI2 | 2.440110328 |  |  |
| VIP | 2.440088252 | * | * |
| SLC4A4 | 2.439841905 |  |  |
| TAP1 | 2.436939837 |  |  |
| LOC105372693 | 2.436302908 | * |  |
| MIR1908 | 2.436064825 |  |  |
| CDC27P10 | 2.434645471 |  |  |
| GAS2L2 | 2.434233174 |  |  |
| LOC105373682 | 2.433898545 | * | * |
| COL8A2 | 2.433385999 |  | * |
| MUC12 | 2.4332528 |  |  |
| NPW | 2.432547581 | * | * |
| LOC107984821 | 2.432508808 |  | * |
| MUC3A | 2.432201501 | * |  |
| LOC317727 | 2.432068583 | * |  |
| LOC105377021 | 2.431110313 |  | * |
| CARD11 | 2.43089317 |  |  |
| LOC105375903 | 2.427665811 |  |  |
| PVALB | 2.427023407 |  |  |
| TSACC | 2.426479919 | * | * |
| NPIPB15 | 2.425755509 | * | * |
| RPL7AP43 | 2.425526206 |  |  |
| ZPLD2P | 2.425278921 |  |  |
| LOC102724527 | 2.424194063 |  |  |
| GAB3 | 2.423736625 |  |  |
| MIR10396B | 2.423076923 |  | * |
| IRAG2 | 2.423052535 |  |  |
| FCMR | 2.420810975 |  | * |
| SYT14 | 2.417800491 | * | * |
| ISX | 2.417419041 |  | * |
| CELA3B | 2.417221171 |  |  |
| KCNMB2-AS1 | 2.4171564 |  | * |
| ITGAX | 2.416850698 | * | * |
| LOC107984432 | 2.415801313 |  |  |
| TXK | 2.415533324 | * |  |
| KRT31 | 2.412407869 |  | * |
| SSC5D | 2.411719941 |  | * |
| TNFRSF12A | 2.411674811 |  |  |
| SNORD116-28 | 2.409847694 | * | * |
| PAN3-AS1 | 2.409041649 |  |  |
| TMEM82 | 2.40845239 |  | * |
| FGF4 | 2.408399793 |  |  |
| HSF4 | 2.407158003 |  | * |
| MIR1302-3 | 2.406424043 |  | * |
| TMEM40 | 2.404880848 |  |  |
| LOXHD1 | 2.403681925 |  | * |
| LOC107986004 | 2.403560016 | * | * |
| SNHG22 | 2.402020882 |  |  |
| DIRAS3 | 2.401023118 |  |  |
| EIF3LP2 | 2.401021145 | * |  |
| DAPK2 | 2.400769404 |  | * |
| GASAL1 | 2.396464524 | * | * |
| CPNE6 | 2.396233033 |  |  |
| FAM90A5P | 2.394784919 |  | * |
| TERC | 2.393509585 | * | * |
| SLCO4A1 | 2.393232754 |  | * |
| LOC100506497 | 2.392099959 | * |  |
| LOC107984256 | 2.391032937 | * | * |
| RPL12P35 | 2.390716247 |  |  |
| LOC106699570 | 2.390158961 |  | * |
| ABCB1 | 2.389323967 |  |  |
| ADAMTSL2 | 2.389306234 |  | * |
| OVAAL | 2.388863818 |  | * |
| SNORD116-7 | 2.387632073 |  |  |
| LMO1 | 2.38744088 |  | * |
| GABRB2 | 2.38430842 |  |  |
| CDH8 | 2.382539068 |  |  |
| PDLIM1 | 2.382342244 |  |  |
| LINC00437 | 2.382278111 |  | * |
| GDF10 | 2.381214602 |  |  |
| SHOX | 2.380058351 | * | * |
| SNORA44 | 2.37997887 |  |  |
| SERINC4 | 2.379264553 | * | * |
| LMNTD2-AS1 | 2.378624326 |  | * |
| ZRANB2-AS2 | 2.375900714 | * | * |
| CBLN2 | 2.375683594 |  |  |
| LOC107986035 | 2.375159687 |  | * |
| TNFRSF10D | 2.375010877 |  |  |
| THBD | 2.37371596 |  |  |
| LOC105378688 | 2.372145723 |  | * |
| CCDC9B | 2.371060025 |  | * |
| LOC105377896 | 2.367915445 | * | * |
| S100A16 | 2.3677564 |  |  |
| LOC102723534 | 2.367175776 | * | * |
| AOC1 | 2.367136109 | * | * |
| TSPAN32 | 2.366247748 | * | * |
| RHOQP3 | 2.366231935 |  |  |
| PNPLA7 | 2.365226713 | * | * |
| TRGV7 | 2.363010318 | * | * |
| CSDC2 | 2.362252849 | * | * |
| PTPRB | 2.361725246 |  |  |
| SLC22A4 | 2.361076838 | * | * |
| LOC105376040 | 2.360824288 | * | * |
| LOC101928368 | 2.359487328 | * | * |
| PTH1R | 2.35826521 |  |  |
| RPSAP5 | 2.357686981 |  |  |
| PVT1 | 2.357226493 |  |  |
| SNORA31B | 2.357142857 |  |  |
| SRGAP2-AS1 | 2.356893995 |  |  |
| LOC112268123 | 2.355261229 |  |  |
| EBI3 | 2.353726044 |  | * |
| CRTAC1 | 2.351718773 | * | * |
| SLIT2-IT1 | 2.351530685 |  |  |
| LOC101929705 | 2.351423295 | * | * |
| LINC01894 | 2.3512502 |  |  |
| C3orf22 | 2.350972589 |  | * |
| LOC101927551 | 2.350564483 |  |  |
| A4GALT | 2.350474976 |  |  |
| SECTM1 | 2.349421244 | * | * |
| TF | 2.349325452 | * | * |
| PTPRN | 2.346587047 |  |  |
| S100A2 | 2.34635972 |  | * |
| ADAMTSL4 | 2.346297814 |  |  |
| GDF15 | 2.345485564 |  |  |
| LOC107984697 | 2.345293897 | * | * |
| MCF2L2 | 2.342950457 | * | * |
| CALB2 | 2.34133087 |  | * |
| RPS20P11 | 2.34032415 |  | * |
| PGF | 2.339616754 | * | * |
| LOC105375228 | 2.3384219 |  |  |
| MFSD6 | 2.33657164 |  |  |
| ASB5 | 2.335921679 |  |  |
| TFPI2 | 2.335443887 |  |  |
| LOC107987175 | 2.334528411 | * |  |
| NKX3-1 | 2.328612804 |  |  |
| RNF207-AS1 | 2.328238007 |  |  |
| ELL2 | 2.32757509 |  |  |
| FN1 | 2.325085665 |  | * |
| LOC105371972 | 2.324683406 | * |  |
| SFTA1P | 2.323967239 |  |  |
| CYP2W1 | 2.322873209 |  |  |
| AMY1C | 2.32193819 |  | * |
| HTR1D | 2.319230769 | * | * |
| FAM87B | 2.31887728 |  |  |
| LOC105371288 | 2.318795871 |  | * |
| BMP2 | 2.318558321 |  | * |
| GALNT5 | 2.315524787 | * | * |
| MIR205HG | 2.315349176 |  | * |
| GJA3 | 2.314801688 |  |  |
| EEF1A1P7 | 2.314157038 | * | * |
| MIR3936 | 2.313146835 |  |  |
| LOC105378808 | 2.312992613 |  | * |
| PHKA2-AS1 | 2.312989362 |  | * |
| ELN | 2.312609723 | * | * |
| LCN10 | 2.312520536 |  |  |
| LOC105371403 | 2.311550323 |  | * |
| LOC105371490 | 2.311167382 |  |  |
| CCM2L | 2.310453098 | * | * |
| GCNT3 | 2.310342152 |  |  |
| POU2F2 | 2.310224108 |  | * |
| APOC1P1 | 2.308316066 | * | * |
| LOC105371485 | 2.306977679 | * | * |
| CBX3P4 | 2.306965064 |  |  |
| C10orf55 | 2.306361247 |  |  |
| MUC20 | 2.304694486 |  | * |
| LOC105379194 | 2.30418866 |  | * |
| KLHL6 | 2.303534164 |  | * |
| SNORD116-12 | 2.301609327 |  |  |
| AMIGO2 | 2.301433772 |  |  |
| RSPH9 | 2.301168478 |  | * |
| LOC100419622 | 2.300092255 |  |  |
| GNRH2 | 2.299979807 | * | * |
| SREBF2-AS1 | 2.299851141 |  |  |
| LRRC32 | 2.297873995 | * | * |
| LOC100129697 | 2.297271144 |  | * |
| RPLP1P4 | 2.295951201 |  |  |
| SEPTIN14P23 | 2.295842217 |  | * |
| DBH-AS1 | 2.294530034 |  | * |
| CCL3 | 2.294424379 |  | * |
| UGT2B7 | 2.293066646 |  | * |
| FAM240C | 2.292759004 |  | * |
| RNU6-890P | 2.291479187 |  |  |
| LOC112268076 | 2.290045789 | * | * |
| LOC105372705 | 2.289061064 | * | * |
| ULBP2 | 2.28717011 |  |  |
| LOC107985725 | 2.28664077 |  |  |
| CREB5 | 2.285693869 |  |  |
| LOC107986361 | 2.284457065 | * | * |
| LOC101928216 | 2.284086509 | * | * |
| ELMOD1 | 2.283399812 |  | * |
| KRT8P45 | 2.282693902 |  |  |
| LOC101930048 | 2.280646037 |  | * |
| HPN | 2.280432186 |  | * |
| AMY1A | 2.278565706 |  | * |
| MIR632 | 2.278267099 | * | * |
| LOC107984257 | 2.276872571 |  |  |
| NPSR1-AS1 | 2.276628484 |  |  |
| LOC107984526 | 2.275981298 |  | * |
| ATP5F1EP2 | 2.275677648 |  | * |
| LINC00638 | 2.274916865 |  |  |
| LOC102723701 | 2.274810118 |  |  |
| PPP1R14A | 2.274336608 |  |  |
| TFAP2A-AS2 | 2.274082741 |  |  |
| HLA-C | 2.271899152 |  |  |
| NEK5 | 2.27171707 |  | * |
| MIR4800 | 2.271547248 |  |  |
| MIR3936HG | 2.270830734 |  |  |
| DPYSL3 | 2.270552391 | * | * |
| CSRP3 | 2.269071138 |  | * |
| ITGA1 | 2.268677885 |  |  |
| RPL7P24 | 2.267009489 | * |  |
| NAP1L2 | 2.266898137 |  |  |
| SSTR2 | 2.266452446 |  | * |
| LMF1-AS1 | 2.266376882 |  | * |
| TLR6 | 2.264206475 |  |  |
| TENT5C | 2.264181953 | * | * |
| KITLG | 2.262921727 | * |  |
| RGS8 | 2.262706322 |  |  |
| LINC01534 | 2.26112324 |  |  |
| SNORD53B | 2.261098554 |  |  |
| EXOC3L4 | 2.260178328 |  | * |
| LOC642975 | 2.260092823 |  |  |
| MIR6747 | 2.259916818 | * | * |
| CPNE4 | 2.259774478 |  | * |
| LOC105373289 | 2.258789296 |  |  |
| LOC107986441 | 2.25852242 |  | * |
| LOC105376299 | 2.257499479 |  |  |
| MEF2B | 2.253800311 |  | * |
| LPAL2 | 2.252178466 | * |  |
| SPINT1 | 2.251425513 | * |  |
| DLGAP1-AS2 | 2.250144922 |  |  |
| NGEF | 2.248626577 |  | * |
| RNA5S2 | 2.248069262 | * | * |
| UPK3B | 2.246596735 | * | * |
| LOC105370765 | 2.245511566 | * | * |
| GGTLC3 | 2.245181182 |  |  |
| SEPTIN14P7 | 2.244170096 |  |  |
| BRI3P1 | 2.243211129 | * | * |
| LOC728533 | 2.243072365 | * |  |
| CLEC4F | 2.241782472 | * |  |
| FSHR | 2.23960258 |  |  |
| MCEMP1 | 2.239308729 |  |  |
| GATA6 | 2.237217561 | * | * |
| LOC105379220 | 2.236548157 |  | * |
| SNORD17 | 2.235219207 |  |  |
| TMEM229B | 2.234782645 |  |  |
| LINC02709 | 2.234634391 |  |  |
| LOC101928174 | 2.234041126 |  |  |
| STAB2 | 2.233979212 |  |  |
| LINC01301 | 2.232541884 | * |  |
| LOC105371632 | 2.23147341 |  |  |
| ADAMTS12 | 2.230471003 |  |  |
| LINC02092 | 2.229326777 | * | * |
| SLC31A2 | 2.228884462 | * | * |
| LOC107986162 | 2.227756089 |  |  |
| LOC105373077 | 2.227381159 | * |  |
| LOC112268267 | 2.226994984 |  |  |
| CLDN4 | 2.225935881 |  |  |
| GADD45A | 2.225507421 |  |  |
| LINC02609 | 2.225497697 |  | * |
| STON1-GTF2A1L | 2.225117884 |  |  |
| KCNJ12 | 2.224708219 |  |  |
| EXTL3-AS1 | 2.224543965 |  |  |
| HLA-DQB1 | 2.223645833 |  | * |
| FAM83G | 2.223603864 |  |  |
| DLGAP1-AS1 | 2.223148298 |  |  |
| TAGLN | 2.222103473 |  |  |
| LINC02167 | 2.22068043 |  | * |
| CAPN9 | 2.220444529 |  | * |
| LOC107984659 | 2.219207271 | * | * |
| IL2RB | 2.217742505 | * | * |
| PLA2G4D | 2.214754877 |  | * |
| LINC00475 | 2.213409965 |  | * |
| EPS8L2 | 2.212624769 |  |  |
| LOC105378988 | 2.212480193 | * |  |
| FAM90A22P | 2.21238321 |  |  |
| P4HA3 | 2.210897983 | * |  |
| CD58 | 2.208922197 |  |  |
| LOC105372700 | 2.207781117 |  | * |
| TMEM144 | 2.207567181 | * |  |
| NPIPB7 | 2.207536083 | * | * |
| REPIN1-AS1 | 2.207451248 |  |  |
| LOC107985050 | 2.207307085 | * | * |
| RNU6-9 | 2.207008596 | * |  |
| MIR4260 | 2.206896552 | * |  |
| LOC105372096 | 2.206483221 |  | * |
| PRRT4 | 2.205732727 |  | * |
| LOC283194 | 2.205227825 |  |  |
| PTPRCAP | 2.204498697 |  |  |
| MIR8072 | 2.20412595 | * | * |
| DNAH17 | 2.203604518 | * | * |
| LOC112268242 | 2.202534221 | * |  |
| RAB15 | 2.202312336 |  |  |
| ARHGAP5-AS1 | 2.202163424 |  | * |
| CCNB3 | 2.201155568 |  |  |
| MUC19 | 2.200550541 |  | * |
| UBTD1 | 2.200081025 |  |  |
| LOC101930370 | 2.199363114 |  | * |
| SEC14L2 | 2.199247613 |  |  |
| CDH16 | 2.197363597 | * | * |
| CRYBB2 | 2.195477688 | * | * |
| PSTPIP2 | 2.195286498 |  | * |
| LOC105374723 | 2.193368698 | * | * |
| APOL4 | 2.192380303 | * | * |
| MISP3 | 2.192362893 |  |  |
| LINC01488 | 2.191205333 |  |  |
| KIRREL1-IT1 | 2.190883334 |  |  |
| FAM230H | 2.190490551 | * |  |
| MYL2 | 2.189980827 |  |  |
| LOC107985201 | 2.189496414 |  |  |
| LOC100128076 | 2.188365621 |  | * |
| SNORD111B | 2.188149703 |  |  |
| LOC107985662 | 2.187176095 |  | * |
| MAFF | 2.186579906 |  |  |
| GPR83 | 2.18656949 |  |  |
| SERPINA5 | 2.183094831 |  | * |
| KLF9 | 2.182029183 |  |  |
| MGAT4C | 2.181480932 |  |  |
| ATP1B4 | 2.180791 | * | * |
| P2RY2 | 2.179623437 | * | * |
| FSTL3 | 2.178919791 | * | * |
| IFI6 | 2.17875679 |  |  |
| DNAH11 | 2.178408548 | * | * |
| SH2D7 | 2.177315073 |  |  |
| SUGCT | 2.177080199 |  | * |
| DAND5 | 2.177073075 |  |  |
| TM4SF19-TCTEX1D2 | 2.17684244 |  |  |
| HSPA12A | 2.176819973 |  | * |
| TMC7 | 2.176276891 |  |  |
| PTX4 | 2.176170558 | * | * |
| MAP3K7CL | 2.175451256 |  |  |
| KCNN4 | 2.175342347 |  |  |
| LOC107984333 | 2.174835911 | * | * |
| LIMK2 | 2.173053298 |  | * |
| AMPD3 | 2.172785372 |  |  |
| TBX21 | 2.172290912 | * | * |
| GALNT16 | 2.172237406 |  |  |
| LOC100505912 | 2.171909246 |  | * |
| RN7SL481P | 2.171280401 |  | * |
| CYGB | 2.171012696 |  |  |
| CRSP8P | 2.17067024 |  |  |
| HTR1E | 2.170656419 |  |  |
| MIR491 | 2.169327898 |  |  |
| SLC6A12 | 2.16918044 | * | * |
| SMIM1 | 2.167135452 | * | * |
| SPATA22 | 2.167122967 |  | * |
| PGR | 2.166954552 |  | * |
| LOC441086 | 2.166306529 |  |  |
| PIWIL4 | 2.16613702 | * | * |
| ELAPOR1 | 2.165643777 |  |  |
| LOC105369339 | 2.164887879 | * | * |
| LOC105379484 | 2.164347363 |  |  |
| HMX3 | 2.164307497 | * | * |
| LRRC38 | 2.163103858 |  | * |
| MIR4477B | 2.162913337 | * | * |
| DDB2 | 2.16220172 |  |  |
| OPN5 | 2.160842038 | * | * |
| EPPK1 | 2.159955371 |  | * |
| ZNF563 | 2.159954886 |  | * |
| AKR1B15 | 2.159327359 |  |  |
| RPS26P3 | 2.15913033 |  |  |
| CACNA2D3 | 2.159115037 |  |  |
| LOC105371044 | 2.154984322 |  |  |
| ADAMTS14 | 2.154102298 |  |  |
| DKK3 | 2.152049002 |  |  |
| NIPAL2 | 2.151821856 |  | * |
| CCDC62 | 2.151236778 |  | * |
| SYN1 | 2.150664777 |  |  |
| GFOD1 | 2.150090881 |  |  |
| LINC02798 | 2.149513491 |  |  |
| C15orf39 | 2.149072889 |  |  |
| KLHL32 | 2.148323153 | * | * |
| LOC101928841 | 2.148058432 |  |  |
| LOC107986593 | 2.147640217 |  |  |
| LOC105374187 | 2.146715995 | * |  |
| ANKRD65 | 2.14634495 |  |  |
| LOC105372270 | 2.145939148 |  |  |
| LOC105374216 | 2.145832241 |  | * |
| GJC2 | 2.144172098 | * | * |
| LOC107984242 | 2.143473371 |  |  |
| RGS5 | 2.143051788 | * | * |
| ITGA3 | 2.142382861 |  |  |
| PLA2G4C | 2.141985685 |  |  |
| CHSY3 | 2.14182067 |  |  |
| PRELID1P6 | 2.141584586 |  | * |
| LINC01465 | 2.14100229 |  |  |
| SNORD3B-2 | 2.137833279 | * |  |
| SNORA59B | 2.135378747 |  | * |
| LOC100129215 | 2.13499328 | * | * |
| VWA5B1 | 2.134183504 |  |  |
| LOC105377572 | 2.133445283 |  | * |
| OTUD7A | 2.132644283 |  | * |
| LAT2 | 2.132626233 |  | * |
| SNORA11G | 2.132044858 | * |  |
| MMP24-AS1-EDEM2 | 2.13106595 |  | * |
| LOC100507156 | 2.130844971 |  |  |
| MICB | 2.130682068 |  |  |
| GPR137B | 2.130571972 |  |  |
| TAGLN3 | 2.129655194 |  |  |
| SLC49A4 | 2.129187994 | * |  |
| LOC112268047 | 2.128838222 |  | * |
| LINC02348 | 2.128243693 |  |  |
| PID1 | 2.127026819 |  | * |
| RPS6KA2-IT1 | 2.126331771 |  |  |
| HRAT92 | 2.125789777 |  | * |
| PRSS47 | 2.125368934 | * | * |
| NPFFR2 | 2.125119246 | * |  |
| CFAP45 | 2.124607086 |  | * |
| ZNF790-AS1 | 2.124198713 |  |  |
| OSTM1 | 2.123829311 |  |  |
| LOC105376958 | 2.123293552 | * | * |
| SPTY2D1OS | 2.122902683 | * |  |
| DNMT3L | 2.12281415 |  | * |
| PAPPA2 | 2.122597101 |  |  |
| TNR | 2.122515073 | * | * |
| LMOD1 | 2.121765241 | * | * |
| RAB6B | 2.121628009 |  |  |
| SLC16A4 | 2.121572125 |  |  |
| DNMT3L-AS1 | 2.121484507 |  |  |
| SLC2A1 | 2.120581766 |  |  |
| MIR6850 | 2.119036041 |  |  |
| PXDC1 | 2.11897904 |  |  |
| TENM3-AS1 | 2.118753858 |  |  |
| LCMT1-AS2 | 2.118607725 | * |  |
| LOC107986845 | 2.118293486 |  |  |
| WNT9A | 2.117754737 |  |  |
| RPL3P6 | 2.117615793 |  |  |
| LOC105370560 | 2.117294641 |  |  |
| ZNF831 | 2.117193698 |  | * |
| GPR12 | 2.115931968 |  |  |
| GRHL3-AS1 | 2.115835135 | * | * |
| LINC02572 | 2.114740881 | * | * |
| IP6K3 | 2.114714089 |  | * |
| LOC105372863 | 2.113649405 |  |  |
| RNF139-AS1 | 2.113510648 | * |  |
| SYNDIG1L | 2.113406014 | * | * |
| MKLN1-AS | 2.11250068 |  | * |
| IMPDH1P5 | 2.111281932 |  |  |
| TRW-CCA1-1 | 2.111174977 | * |  |
| ACTBP2 | 2.108033853 |  |  |
| GDF9 | 2.107352067 |  |  |
| CHST3 | 2.107258463 | * |  |
| KCNE5 | 2.106633686 |  |  |
| CFAP58 | 2.106275912 |  |  |
| PLEK2 | 2.104904676 | * | * |
| SPOCK2 | 2.104131121 |  | * |
| ACTL6B | 2.102525529 |  |  |
| STAB1 | 2.102081464 |  | * |
| LOC100996318 | 2.101061612 |  |  |
| SFXN3 | 2.100807563 |  |  |
| PEX5L | 2.100721564 | * |  |
| FAM133A | 2.099199255 |  |  |
| SCN11A | 2.098518666 | * |  |
| LOC112268048 | 2.098387083 | * |  |
| HERC2P11 | 2.098261817 |  |  |
| FRMD1 | 2.095696697 |  | * |
| DNAH12 | 2.094758065 |  |  |
| TGM2 | 2.093332902 | * | * |
| AGR2 | 2.092929905 |  | * |
| GLRX | 2.092802922 |  |  |
| NINJ1 | 2.092569199 |  |  |
| DCLK3 | 2.091110803 | * | * |
| POU6F2 | 2.089009226 | * |  |
| WDFY3-AS2 | 2.088949158 |  |  |
| CDK2AP2P2 | 2.088701526 |  |  |
| TNC | 2.087712811 |  | * |
| LOC101929003 | 2.086488317 |  |  |
| PLEKHF1 | 2.086049783 |  |  |
| SNORD82 | 2.086043115 |  |  |
| TRH-GTG1-7 | 2.086034932 |  |  |
| NRG3 | 2.085226555 |  | * |
| TLCD1 | 2.084797733 |  |  |
| PRELP | 2.084674427 | * | * |
| LOC105375123 | 2.083950617 |  |  |
| LOC101929367 | 2.083467738 |  | * |
| LINC01750 | 2.082964748 | * | * |
| EPHA2 | 2.082461726 |  |  |
| DKFZP434A062 | 2.082332931 |  |  |
| LOC107986656 | 2.081577548 | * | * |
| HES6 | 2.080241757 |  | * |
| DPP4-DT | 2.080221943 | * | * |
| COL1A1 | 2.080119354 |  | * |
| ITGA7 | 2.079976983 |  |  |
| LOC100288570 | 2.079825454 |  | * |
| LOC107985495 | 2.079274524 |  |  |
| SLC18A3 | 2.078092176 |  |  |
| PRECSIT | 2.077068399 |  |  |
| LOC101929107 | 2.076714655 |  | * |
| PPM1J | 2.076699411 |  |  |
| MIR4324 | 2.075851691 | * |  |
| LOC105379377 | 2.075361351 | * |  |
| SLC43A2 | 2.074686542 | * | * |
| LOC107984895 | 2.073164035 | * | * |
| C14orf178 | 2.071134585 |  |  |
| BTG3-AS1 | 2.070568021 |  | * |
| LOC101928409 | 2.070169516 | * | * |
| IRX4-AS1 | 2.069500562 | * | * |
| RFESD | 2.068387123 |  |  |
| SERTAD4-AS1 | 2.067857866 | * | * |
| ADGRD2 | 2.067027027 |  |  |
| SLC12A4 | 2.065798574 |  |  |
| LPCAT2 | 2.064554558 |  |  |
| RGMB-AS1 | 2.064464781 |  | * |
| PLAAT4 | 2.062535273 |  |  |
| LINC01772 | 2.062318256 | * | * |
| LOC105372236 | 2.06190564 |  | * |
| LRRC73 | 2.057357334 |  | * |
| PRKAG2 | 2.056962328 |  |  |
| PIPOX | 2.05623639 | * | * |
| MYOCD | 2.055642776 | * | * |
| LMX1A | 2.055489075 |  |  |
| RPS26P50 | 2.055373773 |  |  |
| EDIL3 | 2.055315911 |  |  |
| ACTA2 | 2.0542277 | * | * |
| LOC107985303 | 2.054217984 |  |  |
| APOC1 | 2.054200864 | * | * |
| CLCF1 | 2.05408707 |  | * |
| SNORD19C | 2.053756403 | * | * |
| OPRL1 | 2.053147675 |  | * |
| PRCD | 2.053082331 |  |  |
| LINCR-0001 | 2.053060606 |  |  |
| BGLAP | 2.051757062 | * | * |
| ZNF471 | 2.050275663 |  |  |
| BSN-DT | 2.04843872 |  |  |
| SLC7A5 | 2.047795143 |  |  |
| CXCL3 | 2.047486605 |  |  |
| LOC101929494 | 2.045698544 | * | * |
| LOC105374616 | 2.045596871 |  |  |
| LOC101929322 | 2.04487339 |  |  |
| SIK1B | 2.044030922 |  |  |
| FXYD5 | 2.04340425 |  |  |
| KCNQ5 | 2.043201981 |  |  |
| LOC101060187 | 2.040632633 |  | * |
| KCNH1 | 2.040557011 |  |  |
| LOC105374732 | 2.040154998 |  |  |
| PABPC1L2A | 2.040064489 |  | * |
| S100A13 | 2.038868326 | * | * |
| CAVIN2 | 2.038638456 |  |  |
| DIRAS2 | 2.038577134 |  |  |
| MAP6 | 2.03655433 |  | * |
| XKR4 | 2.03536524 |  |  |
| LOC283028 | 2.035104713 |  |  |
| LTBP2 | 2.033873478 |  |  |
| SIK1 | 2.033564702 |  |  |
| SCN4B | 2.033375706 | * | * |
| PDZK1P1 | 2.033017897 | * |  |
| AGXT | 2.03248701 | * | * |
| GADD45B | 2.032352067 |  |  |
| ADORA1 | 2.031607996 |  |  |
| LOC107984984 | 2.030775717 |  |  |
| RAB6D | 2.030414002 | * |  |
| HMOX1 | 2.030267408 |  | * |
| CDKN1A | 2.030087114 |  |  |
| KRTAP10-6 | 2.030080434 |  | * |
| MSNP1 | 2.029008559 |  |  |
| SYT8 | 2.028831896 |  | * |
| RORA-AS1 | 2.02855709 |  |  |
| SPOCK1 | 2.027883482 |  |  |
| MMP11 | 2.026463902 |  | * |
| SIRPAP1 | 2.026416609 |  |  |
| MIR4315-1 | 2.02513465 |  | * |
| POM121L8P | 2.024402537 |  |  |
| SPNS2 | 2.023546713 |  |  |
| CCDC58P4 | 2.023263939 |  |  |
| ZNF710-AS1 | 2.023126836 |  |  |
| LOC642366 | 2.0229721 | * | * |
| LOC107986677 | 2.022823582 | * | * |
| ODAPH | 2.022303499 |  |  |
| LOC102724632 | 2.022002621 |  | * |
| DNAH10 | 2.021833513 |  | * |
| LOC107987292 | 2.021324037 | * | * |
| GATA2 | 2.021323153 | * | * |
| METTL11B | 2.02033494 |  | * |
| MIR6821 | 2.020241647 | * |  |
| GABRD | 2.020129724 | * | * |
| CDC27P9 | 2.019927936 |  |  |
| LOC729732 | 2.019376956 |  | * |
| LOC105377921 | 2.01930389 |  | * |
| CPEB1 | 2.019097173 |  | * |
| MFAP3L | 2.017566832 |  |  |
| LOC102724593 | 2.017215648 |  | * |
| CEMIP | 2.016942971 |  |  |
| MIR940 | 2.016872507 | * | * |
| LOC107987135 | 2.016197953 |  |  |
| C20orf204 | 2.015972458 |  | * |
| PNMT | 2.014735136 | * | * |
| VPS13D | 2.013989516 |  | * |
| BEAN1 | 2.013258968 | * |  |
| ACTR3-AS1 | 2.013112639 | * |  |
| ALS2CL | 2.012853648 |  | * |
| EXTL1 | 2.012733267 |  |  |
| P2RY8 | 2.011972704 |  |  |
| PRSS23 | 2.011381611 | * |  |
| IKZF3 | 2.010997186 |  |  |
| DNAJC22 | 2.009886433 | * |  |
| EIF2S3B | 2.008180591 | * | * |
| GTF2IP7 | 2.007527472 |  |  |
| CBX1P3 | 2.00748782 | * |  |
| ATF3 | 2.006617995 |  |  |
| LOC105374309 | 2.00661271 |  | * |
| SORCS3 | 2.006475482 |  | * |
| FOXL2NB | 2.005851854 | * |  |
| RPS27L | 2.005313648 |  |  |
| RTL1 | 2.005112791 |  | * |
| ADAM19 | 2.004631942 |  |  |
| IFI35 | 2.003775364 |  |  |
| PEAR1 | 2.003328831 |  |  |
| FOSL2 | 2.002983495 |  |  |
| LOC101928402 | 2.002673543 |  |  |
| ITGAD | 2.002649202 | * | * |
| PLP2 | 2.002333672 |  |  |
| DRD2 | 2.001669567 |  | * |
| LOC112268218 | 2.001263679 |  |  |
| AQP7P1 | 2.001124073 |  |  |
| HSPA4L | 2.000496231 |  |  |

The values were presented as fold changes in mRNA level relative to the control (Ctrl). Asterisks indicate genes that increased more than two-fold upon X-ray irradiation and decreased to less than 0.66-fold upon treatment with p38MAPK inhibitors (SB202190 or SB203580).
